# Landscape of G Protein-Coupled Receptor Function from Structure Networks

**DOI:** 10.1021/jacsau.6c00851

**Published:** 2026-07-30

**Authors:** Sara Gentile, Angelo Felline, Sara Mazzali, Francesca Fanelli

**Affiliations:** † Department of Life Sciences, 9306 University of Modena and Reggio Emilia, via Campi 103, 41125 Modena, Italia

**Keywords:** GPCRs, G proteins, structure network analysis, GPCR deorphanization, allosteric communication, allosteric pockets

## Abstract

G protein-coupled receptors (GPCRs) represent the therapeutic targets for an estimated 30–40% of marketed drugs. By translating the majority of the GPCR structures from the Protein Data Bank into structure graphs and analyzing over 2 million structural contacts, this study maps the landscape of GPCR classification and function. A minimal subset of just 30 specific contacts is sufficient to define the signatures of distinct receptor classes, subfamilies, types, subtypes, and functional states. These structural signatures successfully assigned classifications to the orphan GPCRs. Upon activation, class A GPCRs undergo the most radical network reorganization of all classes, retaining 0% of their state-specific contacts when transitioning from the inactive to the active state, a stark contrast to the 32–50% retention observed in classes B1, C, and F. Despite this complete contact turnover across the 7TM bundle, class A activation remains anchored by a highly conserved core of ten universal network nodes. Analysis of representative shortest communication pathways (metapaths) demonstrates that class A GPCRs rely on these highly conserved nodes to bridge structural communication between the orthosteric ligand-binding pocket and the intracellular G-protein-binding site, regardless of the functional state. Furthermore, these metapaths intersect directly with known allosteric binding sites for small allosteric modulators. G protein binding structurally reorganizes the receptor network, funneling a multitude of potential communication pathways into a few preferential routes. These pathways culminate at the highly conserved arginine residue of the E/DRY motif that acts as a key mediator of G-protein recognition, while structural divergences at the receptor-G protein interface dictate the distinctive pathways of specific G-protein signaling. The wide analysis was able to capture key aspects of the structural communication within the GPCR superfamily with implications in drug discovery.

## Graphical abstract

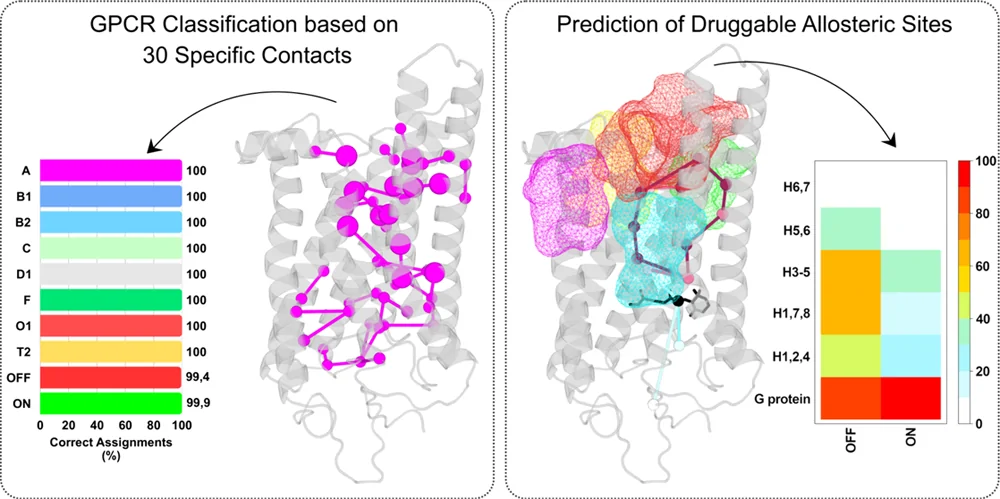

## Introduction

G protein-coupled receptors (GPCRs) form the largest superfamily of receptors in the human proteome, being encoded by more than 800 genes,^1,2^ indirectly dictating a wide array of cellular functions. GPCRs are the targets of a wide variety of ligands and can be broadly divided into two main categories: chemosensory receptors, which bind olfactory and gustatory ligands, chemokines, and chemoattractants; and ‘transmitter’ receptors (also termed endo-GPCRs), which bind retinal chromophores, biogenic amines, (neuro)­peptides, glycoprotein hormones, purine ligands, lipid mediators, and other small molecules such as amino acids and their derivatives.^3,4^ GPCR-mediated signaling pathways have been linked to numerous human diseases, and GPCRs are the targets of an estimated 30–40% of all drugs currently on the market.^5,6^

They share seven-transmembrane (7TM) helices (H) organized in up–down bundle architecture, with an extracellular N-terminus and an intracellular C-terminus (Figure S1). Helices are connected by alternating extracellular (E1, E2, and E3) and intracellular (I1, I2, and I3) loops of different sizes.

They can be classified into ten major families (classes): class A, rhodopsin; class B1, secretin; class B2, adhesion; class C, glutamate; class D1, Ste2-like fungal pheromone; class E, cyclic AMP receptors; class F, frizzled; class O1, fish-like odorant; class O2, tetrapod-specific odorant; and class T2, taste 2; class E lacks high-resolution structures (https://gpcrdb.org/ ^7–11^ ). While sharing the same architecture and topology (fold), each class shows its own peculiarities (Figure S2). Class A–Rhodopsin is the most abundant, comprising over 85% of GPCR genes. In class A GPCRs, almost all the 7TM-helices hold structural irregularities essentially due to the presence of conserved proline residues, which contribute to bulges (H4 and H5), kinks (H6), and deviation from the canonical α-helical structure (H7). Class B1 is characterized by a funnel-shaped enlargement in the extracellular half; such a peculiar shape is more evident in the active states than the inactive ones.^12^ Adhesion/class B2 GPCRs (aGPCRs), the second-largest class of GPCRs, are characterized by a very large extracellular domain, containing the conserved GPCR autoproteolysis-inducing (GAIN) domain.^13^ All but two aGPCR structures in the protein structure databank (PDB) are in the active states in complex with heterotrimeric G proteins and hold the tethered agonist that occupies the extracellular half of the helix-bundle. Among the aGPCR structures solved to date, the GAIN domain covalently linked to the 7TM bundle is resolved in only a single structure: the inactive state of the aGPCR CD97 (PDB: 8IKJ).^14^

Family C GPCRs exist and work as constitutive homo/heterodimers. Class C GPCRs function as constitutive homo- or heterodimers. On average, the 7TM helices of class C receptors are shorter and exhibit fewer deviations from the canonical α-helical structure than those of class A GPCRs (Figure S2).^15^ They hold a huge extracellular domain (ECD), which includes the ligand-binding Venus flytrap domain (VFT) followed by a cysteine-rich domain (CRD) linker connected to the 7TM-helix bundle.^15^ The class D1 structures released so far in the PDB concern the *Saccharomyces cerevisiae* pheromone receptor Ste2, in an antagonist-bound inactive state and an agonist-bound active state coupled to the heterotrimeric G protein Gpa1-Ste4-Ste18. In the Ste2 structure, H4 is shifted by more than 20 Å and the G protein-binding site is a shallow groove rather than a cleft. Ste2 was purified as a homodimer, whose interface involves the N-terminus, E1, H1, H2, and H7 (Figure S2).^16^ Class F GPCRs are characterized by large and structured extracellular regions including the N-terminus, which are all involved in disulfide bridges.^17^ Class O1 is class A-like (https://gpcrdb.org/), sharing with rhodopsin-family GPCRs all highly conserved amino acids, the same irregularities and orientations of the seven-helices, and the same conformational changes associated with activation.^18,19^ Indeed, the odorant receptors used to be class A members.^18,19^ Finally, as for class T2, distinct features include a different structural arrangement of H3, H4, and H5 that forms a pocket near the membrane bilayer. Furthermore, the conformation and reciprocal orientations of H6 and H7 differs significantly in the various receptor types; specifically, I2 forms a short helix oriented toward the membrane surface.^20–22^ In all GPCRs, helices H1, H2, H3, and H5 are tilted, whereas H4 exhibits a similar tilt exclusively in class D1. Across all classes, H3 is the most deeply buried helix within the transmembrane bundle, a feature shared by H7 in classes D1 and T2 (Figure S1). Furthermore, H7 is typically followed by a cytosolic amphipathic helix (H8) that runs parallel to the membrane plane.

The most divergent subfamilies belong to class A, and those with at least one representative in the data set include: alicarboxylic acid, amine, lipid, nucleotide, peptide, protein, and sensory GPCRs (Figure S3). In class A GPCRs, the major portion that distinguishes the subfamilies is E2, which is in (a) random conformation in most amine and steroid receptors and in some lipid, nucleotide, and protein (i.e., the glycoprotein hormone) receptors; (b) variously oriented α-helix in some amine (i.e., β-adrenergic receptors (AR)) and nucleotide (i.e., the adenosine receptors) receptors; and (c) variously oriented of the β-hairpin in alicarboxylic, melatonin, peptide, and sensory receptors, the majority of protein receptors, and some lipid receptors. In peptide and, to a greater extent, most protein GPCRs, H7 and, to a lesser degree, H6 extend into the extracellular side. There, they swing outward from the 7TM core, thereby enlarging the orthosteric-binding cavity (Figure S4).

Originally, transmitter GPCRs were considered orphans because their ligands were unknown.^3,23^ Since the mid-1980s, deorphanization has relied on ‘reverse pharmacology’, targeting exogenously expressed orphan GPCRs with potential transmitters, and was accelerated until the early 2000s by high-throughput screening of thousands of compounds.^3,23^ Fully deorphanizing a receptor also requires understanding its physiological and clinical roles, a process recently aided by single-cell RNA sequencing.^24^ Today, reverse pharmacology is integrated with high-throughput signaling assays that leverage genomic, transcriptomic, metabolomic, and peptidomic data.^24^ Examples include combining yeast-based screening with ligand docking,^25^ screening gut microbiota metabolomes against the nonolfactory GPCRome,^26^ and using bioinformatics, machine learning, and comparative genomics within the peptidergic system.^27^ The latter approach used comparative sequence and structural analyses to map the human peptide-receptor signaling landscape, mining the human genome to successfully pair potential endogenous peptide ligands with five orphan GPCRs.^27^ Similarly, a library of predicted *N. vectensis* neuropeptides was screened against selected N. vectensis GPCRs, identifying 31 neuropeptide receptors.^28^ However, high-throughput approaches remain limited by high costs and frequent ligand–receptor pairing failures due to complex GPCR signaling.^24^

The active states of GPCRs stabilized/induced by various entities (e.g., photon, ions, small molecules, peptides, proteins) couple and activate the α-subunit in heterotrimeric G proteins, which are members of family α in the Ras GTPase superfamily. In Ras GTPases, nucleotide switch and hydrolysis are performed by the conserved core, Ras-like domain, or GTPase (G) domain, which represents the basic functional unit of the G protein, being deputed to GDP/GTP binding and the universal switch mechanism.^29^ The G domain holds a Rossmann fold, which is characterized by a 3-layer (αβα)-sandwich architecture, contributed by five parallel strands (S1 and S3–S5) forming a sheet packed against five helices: H1 and H5 on one side and H2–H4 on the other side (Figure S5). Two linkers connect the G domain to the helical (H) domain, which is an orthogonal bundle of six helices (HA-HF) (Figure S5). The process of receptor-catalyzed nucleotide exchange involves a detachment of the H domain from the G domain.^30^ In that respect, the GPCR acts as a Guanine Nucleotide Exchange Factor (GEF).^1^

Despite the marked divergences in the mode of extracellular agonist binding, the majority of GPCR active states, particularly those of classes A, B1, B2, and O1, share the opening of a cytosolic crevice between H3 and H6, which accommodates the C-terminal helix of the Gα protein. In Classes C, D1, F, and T2, the C-terminal helix of the G protein interacts with I2, I3, and the cytosolic extensions of H5 and H6 (Figure S6). The GPCR-G protein complexes in the PDB comprise essentially five signaling divergent G proteins: Gi/o, Gs, Gq/11, G12/13, and Gt.

Significant advancements in GPCR structure determination have facilitated the use of enhanced-sampling molecular simulations to study ligand binding, receptor activation, and intracellular protein coupling.^31–40^ Notably, an extensive simulation of the β2-adrenergic receptor (β2AR) using Google’s Exacycle platform aggregated thousands of independent trajectories into a Markov state model, totaling two milliseconds of dynamics. This study demonstrated that β2AR samples multiple discrete conformational states, with active and inactive states connected by distinct pathways that are selectively modulated by different ligands.^33^

While most studies focus on a single GPCR, the Protein Data Bank (PDB) contains hundreds of structures across diverse classes, subfamilies, and functional states. Addressing this diversity, a large-scale simulation study^41,42^ analyzed 284 GPCRs without coupling partners, spanning 35 types across four major classes (A: 90.14%, B1: 4.23%, C: 2.11%, and F: 3.52%). By tracking the distance between specific positions in helices H2 and H6, a known marker for the outward motion of H6 during class A and B1 activation, the authors characterized closed, intermediate, and open conformational states. The trajectories revealed local breathing motions driving closed-to-intermediate and closed-to-open transitions.^41^

Interhelix distances were established as hallmarks of active and inactive GPCR states also in another study.^43^ When calculated across 16 different systems (four GPCRs in various states over 500 ns trajectories), these distances were integrated into an ‘activation index’ ranging from – 20.4 to 20.7 for inactive states and 8.1 to 110.2 for active states.^43^

A large-scale analysis of structural communication in the GPCR superfamily is missing. To explore the landscape of GPCR function, hundreds of GPCR structures can be merged into a large conformational ensemble and analyzed using graph-based methods such as Protein Structure Network (PSN) analysis, capturing structural communication. Representing biomolecular structures as networks of interacting amino acids or nucleotides is increasingly used to elucidate complex phenomena and is particularly suited for investigating how GPCRs transfer extracellular signals to cytosolic partners.^44–75^

We implemented PSN analysis in the webPSN server^60,71^ and the standalone Wordom and PSNtools software.^74,76^ Within this framework, we built the psnGPCRdb database (http://webpsn.hpc.unimo.it/psngpcr.php), which stores the structure networks (linked nodes, hubs, communities, and communication pathways relative to single, difference, and consensus networks) of all updated GPCR structures in the PDB, both in isolated states and in complex with other molecules.^75^ A unique feature of this approach is that, for each structure, pseudo Cα-atom trajectories generated by Elastic Network Model-Normal Mode Analysis (ENM-NMA) are used to infer the internode correlated motions required to compute reliable communication pathways.^74^ The ENM-NMA approach incorporates the dynamics into single structures.^77^ ENM is a coarse-grained description of protein structures based on Cα-atoms interacting via a Hookean harmonic potential. This model relies on the fact that global mode shapes are dominated by the network of inter-residue contacts, a purely geometric quantity defined by the protein’s overall native topology. Cartesian pseudo Cα-atom trajectories can be determined by triggering normal mode deformations.^78^ ENM-NMA has significantly improved our understanding of the collective dynamics of various allosteric proteins.^77,79,80^

Here, we combined the structure networks of nearly all PDB-deposited GPCRs with ENM-NMA to identify the structural and dynamic determinants that govern GPCR clustering into functional states, signaling states, classes, subfamilies, types, and subtypes. We found that a small minority of specific contacts capture the unique signatures of these different receptor groupings. These signatures were then leveraged to assign class and subfamily to the orphan GPCRs within the data set. Ultimately, this unprecedentedly broad analysis captures fundamental aspects of structural communication that dictate the function across the GPCR superfamily.

## Methods

### Structural Model Preparation, Considered Data Sets, and Allosteric Binding Site Prediction

1728 GPCR structures (2025–08–27 being the latest release date, (Table S1)), were manipulated using the latest release of our Wordom^81^ software. Alternative locations were solved and only one biological assembly was selected if more than one was present. Missing or incomplete side chains were added and automatically modeled in their rotameric states, while relieving bumps surrounding amino acid residues, according to the following automated iterative procedure. At step 1, missing/incomplete side chain-heavy atoms are identified. At step 2, missing/incomplete side chain-heavy atoms are added using the Dunbrack rotamer library^82^ available in UCSF Chimera.^83^ At step 3, only added atoms, i.e., original atoms are held fixed, undergo energy minimization by MMTK routines^84^ using the Amber and Antechamber (for nonstandard residues) force fields.^85^ The employed routine comprises a steepest descent minimization step to relieve highly unfavorable clashes, followed by a slower but more effective conjugate gradient minimization step. At step 4, inter- and intraresidue atomic clashes of modified amino acids are checked, identifying all pairs of noncovalently bound atoms below a cutoff of 1.95 Å. At step 5, if one or more bad contacts are identified, a new round of energy minimization is performed on the whole modified side chains, i.e., not only on added atoms. The algorithm ends if no more clashes are found. At step 6, if bad contacts are still present after step 5, all atoms but the β-carbon of the modified side chains are deleted, and the algorithm starts from step 2; otherwise the algorithm ends. At step 7, if modified residues are still involved in bad contacts, a final energy minimization step on both side-chain and backbone atoms of completed amino acids is done.

For handling the structure data set in a comparative context, i.e., computing consensus and difference structure networks, positional labeling, such as the generic residue numbering for GPCRs, G proteins, and arrestins, was employed (https://gpcrdb.org/protein/ ^7–10^ ). Specifically, the positional labeling employed for GPCRs is such that the digits before “x” indicate the receptor portion (i.e., helix or loop), whereas the digits after “x” indicate the amino acid position in the considered portion. The position is numbered relative to the most conserved amino acid in the considered portion in class A GPCRs that is given the number 50.^7–10,86^ The positional labeling for the Gα proteins is composed of three dot-separated fields: the capital letter indicating the domain (G or H), the name of the secondary-structure element, and the progressive amino acid position in the secondary structure element.^7–10,87^ The loops that connect two secondary structure elements are indicated by the lowercase names of the connected regions.

The orthosteric ligands (e.g., ions, small molecules, peptides, and proteins) received the same label. Allosteric ligands were also labeled according to their allosteric sites. In those cases, in which the ligand is a peptide or a protein, after computing the contacts involving the ligand, all ligand amino acid residues received the same label, thus being treated as a single node involved in all intermolecular contacts. In general, positional labeling serves to treat structurally equivalent residues in different systems as if they were the same residue. All the variable regions of the receptors, e.g., loops (except for specific structurally conserved loop positions, which could receive positional labeling) and N-terminal and C-terminal tails were condensed in a single node bearing the name of the receptor portion.

Amino acid conservation was computed as well by a locally implemented version of the ConSurf routine,^88^ using 20–95% and 35–95% sequence identity ranges for GPCRs and all other intracellular proteins, respectively. The conservation grades (i.e., integer numbers from 1 (variable) to 9 (highly conserved)) of each receptor amino acid in the structures were averaged over the members of a given receptor hierarchy: subtype → type → subfamily → class. The average conservation values listed in Table S2 concerned only the 7TM-helices and were rounded to the nearest integer.

The whole data set comprised class A, B1, B2, C, D1, F, O1, and T2 structures, with class A accounting for 77.9% of structures. At least one representative from each of the class A subfamilies was present, with the peptide and amine subfamilies covering 33.2% and 26.4% of class A structures, respectively. The whole set of non-orphan GPCRs was used to develop a method for inferring the signatures of GPCR classifications and for assigning class and subfamily to orphan receptors.

The allosteric communications intrareceptor and between receptor and G protein were, respectively, investigated on two distinct nonredundant subsets of ligand-bound structures: one made of 390 class A structures comprising 145 inactive and 245 active states, i.e., functional-state-MP set, and the other made of 202 active-state structures, i.e., signaling-set. As also done for the functional-state set employed to infer specific contacts, only GPCRs in complex with G proteins were selected as representatives of the ON states. The signaling-set was limited to complexes with Gi/o (151 structures) and Gs (51 structures), since those with the other G proteins were too few. The complexes were assigned to the generic Gi/o and Gs signaling pathways depending on the sequence of the α-subunit, excluding the G.HN helix. The G protein chimeras at the C-term were excluded because they could not be unambiguously assigned to a signaling pathway. The two sets comprise members of the alycarboxylic acid, amine, lipid, nucleotide, peptide, protein, and sensory subfamilies. In both sets, the selected active states are represented by the GPCR in complex with the ligand and at least the whole Ras-like domain of the G proteins Both sets were paired, respectively, with test sets made of 91 and 48 structures.

The GPCRs in the functional-state-MP set underwent prediction of potential ligand binding sites in allosteric communication with the orthosteric site by the updated version of the CavityPlus server.^89^ Prediction of the allosteric communication relied on the latest version of the CorrSite2.0 method, which predicted the allosteric sites on the basis of Gaussian network model-based correlations between allosteric sites and orthosteric sites. Only the binding sites characterized by strong druggability and correlated (i.e., with a coefficient ≥ 0.5) with the orthosteric site were considered in the analysis. The amino acid residues contributing to each “allosteric” cavity in the GPCR subset were merged, and only those contributed by at least 70% of structures were taken to define the allosteric site. Allosteric cavities in the OFF and active ON states were taken separately.

All structural analyses were carried out by the in-house software Wordom^76,81^ and PSNtools.^74^

### Computational Approach: PSN Analysis

PSN analysis is a product of the graph theory applied to protein structures, based on the approach described by Vishveshwara and co-workers.^45,90^

In detail, a graph or network is defined by a set of nodes connected by links (contacts). In a PSN, each linked residue (e.g., amino acid, nucleotide, small molecules, ion, etc.) is a node.^91^ Contacts (more technically defined as links) form if the noncovalent interaction strength between pairs of nodes equals or overcomes a cutoff (*I*_min_). Such interaction strength, expressed as a percentage, is computed by the equation below:

$$I_{ij}=\frac{n_{ij}}{\sqrt{N_{i}N_{j}}}\times 100$$

where *I*_*ij*_ is the percentage interaction between residues *i* and *j*; *n*_*ij*_ is the number of heavy atom–atom pairs between the side chains of residues (only for glycine residues the Cα-atoms are considered) *i* and *j* within a distance cutoff (4.5 Å); and *N*_*i*_ and *N*_*j*_ are normalization factors for residue types *i* and *j*, which account for their propensities to make contacts with surrounding residues.^45,90^

As for the normalization factors, both the PSNtools software and the webPSN server employ an internal database holding the normalization factors for the 20 standard amino acids and the 8 standard nucleotides (i.e., dA, dG, dC, dT, A, G, C, and U), as well as for 41500 molecules (e.g., small molecules, lipids, sugars, etc.) and ions extracted from all the structures deposited to date in the Protein Data Bank (PDB). Normalization factors are computed as described in the relevant paper by Kannan and Vishveshwra.^92^ In detail, the normalization factors for the 20 standard amino acids (*N*_*r*_) were computed on a nonredundant data set of proteins with resolution higher than 2 Å, according to the following formula:

$$N_{r}=\frac{\overset{p}{\underset{k=1}{\sum }}\max\left(r_{k}\right)}{p}$$

where *r* is the residue type and *k* is the considered protein. The number of interaction pairs (i.e., the number of atom–atom pairs within 4.5 Å, considering both main-chain and side-chain) made by residue type *r* with all its surrounding residues in a protein *k* was evaluated. The maximum number of interactions made by residue type *r* in protein *k*, represented by max­(*r*_*k*_), was computed for each protein *k* in the data set. The final normalization factor for each amino acid residue type is the average of the maximum interaction value of residue type *r* over the whole data set of proteins *p*, in which residue type *r* occurs.^92^ Accordingly, the normalization factor for a nonstandard amino acid residue (hereinafter referred to as nsr for sake of brevity) is defined as the number of interaction pairs made by the nsr with all surrounding atoms, averaged over the total number of PDB structures, in which that residue is present. If a given nsr is present more than once in the same PDB file, the maximum number of contacts is considered for calculating the average. When a PDB file is submitted, the software automatically retrieves all the normalization factors from the internal database and, if an unparameterized nsr is present, it transparently calculates the normalization factor of the new residue, by applying the method described above to the submitted coordinates.

Thus, the interaction strengths (*I*_*ij*_) are computed for all node pairs. At a given interaction strength cutoff, *I*_*min*_, any residue pair *ij* for which *I*_*ij*_ *≥ I*_*min*_ (see eq 1) is considered to be interacting and hence is connected. Those residues making zero edges are termed as orphans, and those that make at least four edges are referred to as hubs at the considered *I*_*min*_. The four-contact cutoff for hub definition relates to the intrinsic limit in the possible number of noncovalent contacts made by an amino acid in protein structures, due to steric constraints, and it is close to its upper limit. Most amino acid hubs indeed make from 4 to 6 contacts.

The *I*_*min*_ cutoff is set automatically according to the size of the largest node cluster. In detail, a cluster is an ensemble of nodes connected by at least one contact. As for cluster identification, nodes are clustered together using an agglomerative clustering method based on a single-linkage-like criterion. In the beginning, each node is in its own cluster and clusters are then iteratively merged into larger clusters. At each step, two clusters are merged together if there are at least two nodes, one per cluster, with an interaction strength ≥ *I*_*min*_. The process is repeated until no more merging can be performed.

According to the study by Brinda and Vishveshwara on a set of 200 size-divergent protein structures, irrespective of protein size or fold, the normalized size of the largest cluster (in terms of number of nodes) in each protein undergoes a transition at a particular *I*_min_ value named *I*_critical_.^45^ The *I*_critical_ is therefore the *I*_min_ value at which the size of the largest node cluster at *I*_min_ = 0% halves.^45^ In our PSN analyses, the *I*_min_ cutoff is automatically set equal to the *I*_critical_ approximated to the second decimal place. To avoid excessive network fragmentation, which would impair the search for shortest communication paths (see below), all clusters are iteratively connected by the contact with the highest sub-*I*_critical_ interaction strength.

Whereas clusters are ensembles of nodes involved in at least one contact, node communities are densely linked portions of the network. Communities consist of fully interconnected sets of nodes so that intracomunity nodes are densely linked between each other but poorly linked with nodes outside the community. Community building consists of merging sets of three fully interconnected nodes (i.e., *k* = 3-cliques) that share at least one contact.

A meaningful way to exploit PSN analysis is prediction of allosteric communication between distal sites by computing communication pathways through the structure network. A pathway describes how signals are transferred between sites and consists of a set of residues in dynamic contact.^46,93^ Allosteric communication depends on structure and correlated motions. Therefore, to infer the allosteric communication in a system, the PSNtools software searches for the shortest pathways between all residue pairs (path extremities) while accounting for correlated motions, i.e., collective structural fluctuations. Briefly, the procedure consists in searching for the shortest pathways between all node pairs (path extremities). Output pathways are then filtered to retain only the ones, in which at least one internal node holds correlated motions (i.e., with a correlation coefficient ≥ 0.7) with one of the two path extremities. Computation of correlated motions on a single structure, which is the case of psnGPCRdb, is done by ENM-NMA.^94^ Filtered paths can be used to compute metapaths made of the most recurrent (i.e., with a recurrence ≥ 10) nodes and contacts in the path pool. A metapath provides a coarse/global picture of the whole structural communication in the considered system.

PSNtools allows comparisons of two networks or computations of consensus networks on sets of homologous and/or analogous macromolecular structures or conformational ensembles. Network comparisons and consensuses serve, respectively, to infer differences in functionally different states of the same system or network-based signatures in groups of biomacromolecules sharing either the same functionality or the same fold. Consensus networks are made of those links holding average interaction strengths higher than an automatically set average interaction strength cutoff (i.e., an average *I*_min_ ^74^). Difference networks highlight nodes and links shared by the two networks or peculiar to either one of the two networks. Consensus networks can be used to infer metapaths representative of a given aggregation of receptors, which we named consensus metapaths.

### Computation of Specific Contacts

All receptors, except orphan and unclassified/viral receptors, were organized in a four-level hierarchy consisting of class, subfamily, type, and subtype. This hierarchy was used to calculate the average structural networks of each possible receptor aggregation belonging to the same hierarchy level, through a bottom-up approach. For example, the average structural network of subtype A_1_ receptors is computed by averaging the interaction strength of all receptor-receptor and receptor-orthosteric ligand contacts present in the structure networks of these receptors. This process is repeated by going up the levels of the receptor hierarchy, using at each step the set of average networks calculated in the previous step, until arriving at the class level, according to the scheme: subtype → type → subfamily → class. For example, the average structure networks of receptors A_1_, A_2A_, A_2B_, and A_3A_ (hierarchy level subtype) were used to calculate the average structural network of adenosine receptors (hierarchy level type), the latter was then used to calculate, together with the average structural network of P2Y receptors, the average structural network of nucleotide receptors (hierarchy level subfamily), which, in turn, contributed to form the average structure network of class A (hierarchy level class) together with all the other average structure networks of the receptor subfamilies of this class.

Links in each average structure network were ranked based on a relevance score that indicates how different the average interaction strength of the link is from the strength the same link has in all other average structure networks in the same hierarchy level. This score was calculated using the following formula):

$$l_{s}=\frac{\overset{N}{\underset{n}{\sum }}f_{A}-f_{n}}{N}$$

where *l*_*s*_ is the score assigned to a given link *l* in a given average structure network *A*, *f*_*A*_ and *f*_*n*_ are the average interaction strengths of link *l* in network *A* and in the *n* ^th^ network, respectively, and *N* is the total number of networks in the same hierarchy of *A*.

Finally, links were sorted by descending score, and the first links with the highest score (30 links in this case study) were selected as contacts specific to a given average-structure network.

Computation of specific links shared by at least two receptor aggregations in the same hierarchy level focused only on those links involving at least one node with a positional label.

Each orphan receptor was assigned to the class/subfamily with the highest similarity calculated using the following formula:

$$S_{o,n}=\frac{\overset{30}{\underset{l-1}{\sum }}\sqrt{{\left(n_{l}-o_{l}\right)}^{2}}}{30}$$

where *S*_*o,n*_ is the similarity score between the orphan network *o* and a given average structure network *n*, respectively, *l* is one of the 30 most peculiar links in the average structure network *n*, and *o*_*l*_ and *n*_*l*_ are the interaction strengths of link *l* in the structural network of orphan receptor *o* and the average structure network *n*, respectively.

## Results

### Specific Contacts Encode the Signatures of Receptor Classification and Function

Comparisons of structure networks averaged over different aggregations of homologous proteins were applied to the GPCR superfamily to infer those contacts specific to each aggregation. Network consensus and comparisons require positional labeling of any residue in the network, which treats structurally equivalent residues in different systems as if they were the same residue (Methods).

The strengths of contacts were used to build average networks at different GPCR hierarchy levels, e.g., subtype, type, subfamily, class, and functional state. Contacts in each average structure network were ranked based on a relevance score that indicates how different the average interaction strength of a given contact is from the interaction strength of the same contact in all other average structure networks in the same hierarchy level. The first 30 contacts with the highest score turned out to be enough to mark a given hierarchy, i.e., “specific contacts”. Network analysis was carried out on a data set of 1728 GPCR structures (2025–08–27 being the latest release date, (Supplementary Table 1 (Table S1)), including 160 orphans. Nonorphan GPCRs in the set (1568 structures) divide in eight classes: A, B1, B2, C, D1, F, O1, and T2 (https://gpcrdb.org/protein/ (Figure S2)). The majority of them (77.9%) belong to class A. The hierarchies that concern the GPCR functional states, i.e., inactive (OFF) and active (ON), were analyzed as well in a subset of 1240 GPCRs (functional-state set) containing non-APO and, for the ON states, G protein-bound forms (352 OFF and 888 ON states (Table S1)). All physiologically relevant components of the structural models were included in network calculations (annotated in Table S1).

Each GPCR in the nonorphan and functional-state sets was reassigned to class, subfamily, type, subtype, and functional state on the basis of the highest similarity between the strength of the 30 specific contacts in the considered receptor and the average strengths of the same contacts in a given aggregation. Almost all GPCRs in the nonorphan set, independent of the functional state, could be correctly assigned to a class (100%), subfamily (99.7%), type (99.6%), and subtype (94.4%) (Figure 1A).

**Figure 1:**
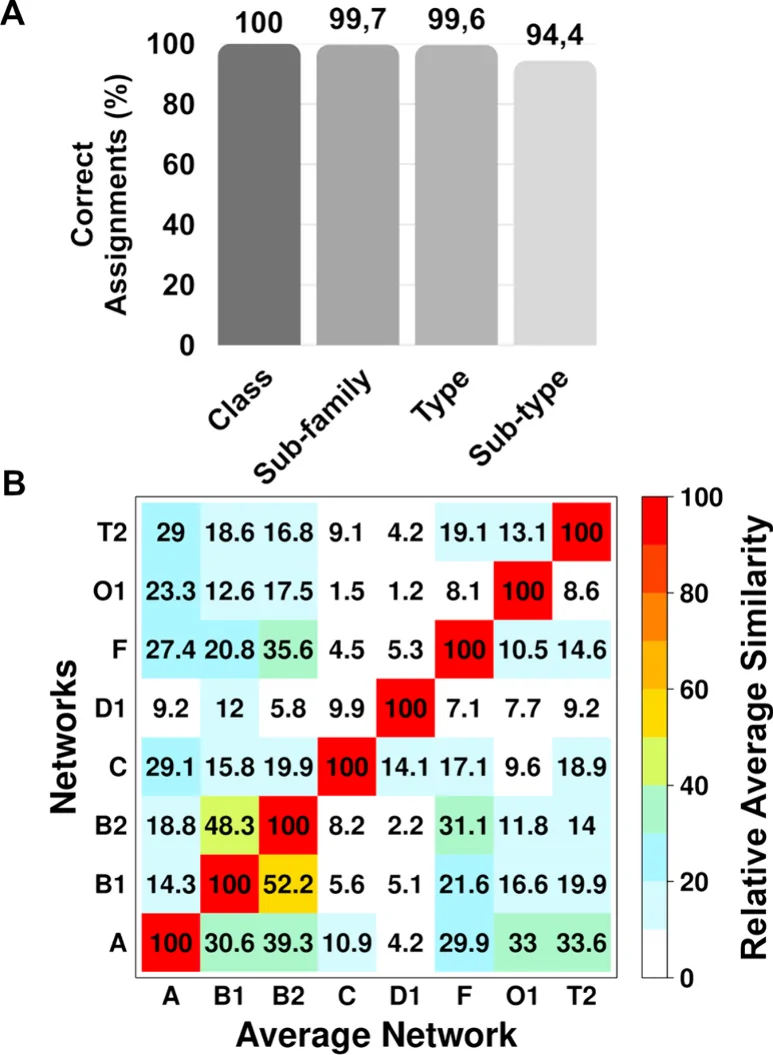
**GPCR assignments to class, subfamily, type, and subtype based on specific contacts.** (A) The percentages of correct GPCR assignments in the different aggregations are shown. (B) The matrix shows the relative average similarity (as percentages) between the specific contacts in the average networks of the GPCR classes (columns) and the contacts in the single networks in the same aggregation (rows). The pairwise comparison with the highest similarity is set to 100.

Class-specific contacts are distributed throughout the entire receptor structure, primarily involving nodes located on helices (Tables S2A and S3A and Figures 2, S7, and S8A). For class A, the most and least involved helices are H3 and H1, respectively. Using class A as a reference, the relative contribution of H5 tends to decrease for most other classes (with class B1 being an exception), while the contributions of H1 and H2 tend to increase, and that of H7 remains highly variable. Finally, the contribution of H6 is almost halved for Classes D1 and T2, whereas it doubles for class F, due in part to the exceptional extension of this helix (Figures 2, S7, and S8A). Noticeably, the presence of the G protein’s G domain docked onto only one protomer of the class C dimer alters the distribution of specific links compared to the other protomer (Figure S7). Specifically, these links tend to shift from the cytosolic to the extracellular half when moving from the G-protein-bound (left) protomer to the G-protein-free (right) protomer. In contrast, such a marked asymmetry in link distribution does not occur in the D1 dimer, likely because both protomers are bound to at least the H5 helix of the G protein (Figure S7). This provides evidence that G-protein binding directly impacts the structural network of the receptor.

**Figure 2:**
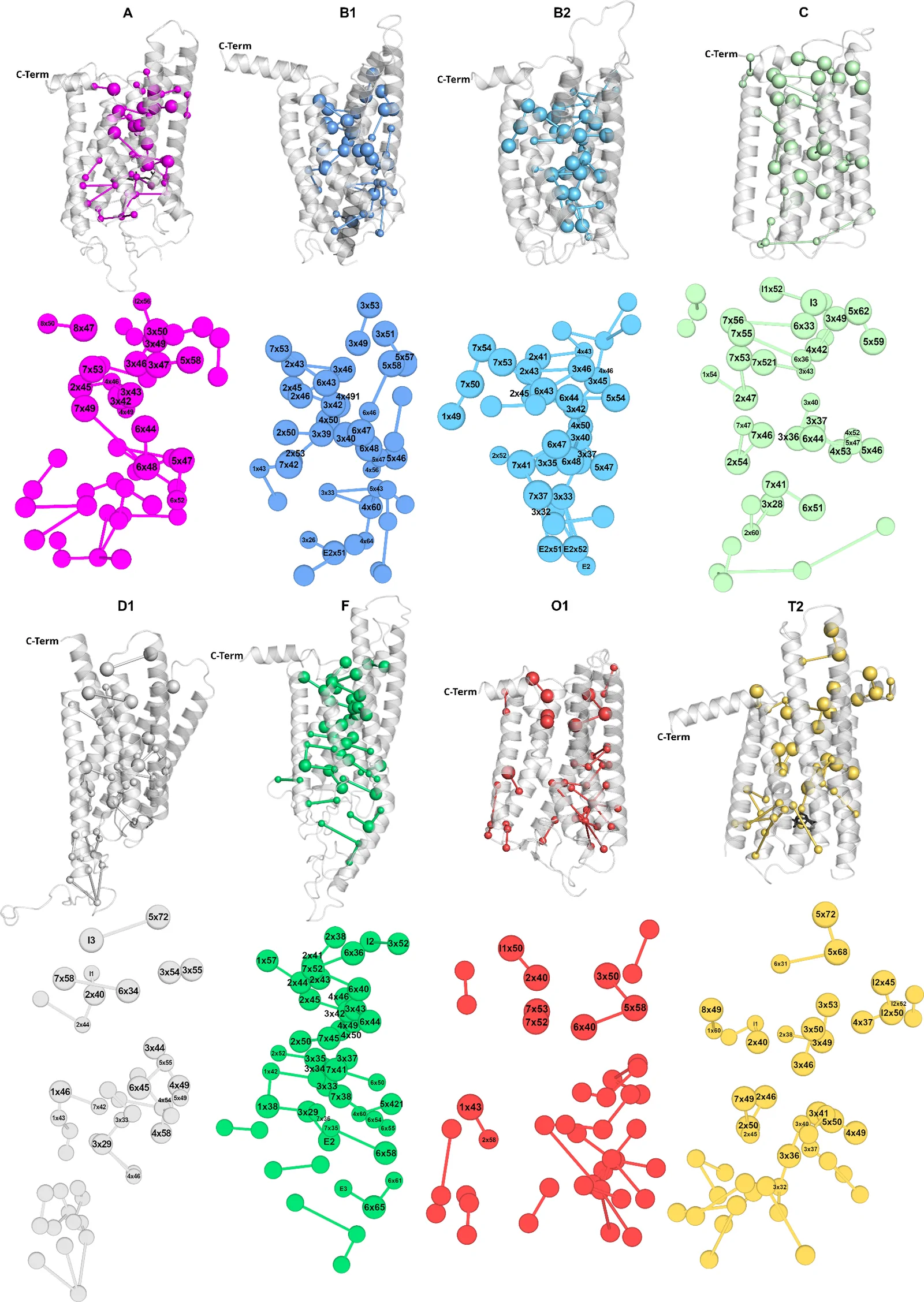
**Visualization of class-specific links.** class specific contacts are mapped onto representative 3D structures of GPCR classes in their active states: class A, 6OYA; B1, 6X18; B2, 7WU5; C, 7EB2; D1, 7AD3; F, 6XBL; O1, 8F76; T2, 7XP6. Cartoon representations of the receptors are shown from a perspective perpendicular to their main axis, with the cytosolic side at the top. The biggest spheres concern the highly conserved nodes (characterized by average ConSurf conservation grades ≥ 8). Only those nodes and their linked partners are labeled. For the C and D1 classes that are constitutive dimers, only the protomer bound to the G domain of the G protein is shown. For classes B1, C, and F, some portions of the extracellular regions and of the peptide agonist (of B1) are not shown.

The degree of evolutionary conservation of specific contact-involved nodes in the 7TM-helices varies in a class-dependent manner. Indeed, conserved nodes (i.e., with average ConSurf^88^ conservation grades ≥ 7) are above 80% for classes B1, B2, C, and F, and below 59% for classes A, D1, O1, and T2 (Tables S2B and S4, and Figure S8B). Noticeably, by considering all classes together, 57.6% of specific contacts (144 out of 250) involve highly conserved positions (with average ConSurf conservation grades ≥ 8), whose distribution in each helix is 5 in H1, 11 in H2, 24 in H3, 9 in H4, 14 in H5, 15 in H6, and 15 in H7, thus highlighting the relevance of H3.

By considering both the full- and active-state data sets (Figure S9), the helices that contribute most significantly to class-specific contacts are H3 for classes A, B1, B2, and T2; H7 for class C; H1 and H3 for class D1; H3 and H6 for class F; and H5 and H6 for class O1.

Only 7.6% (21 out of 275) of the specific contacts are shared by at least two classes. Class B1 shares the maximal number of contacts (10) with the other classes, mainly with B2 (Table S3A). By comparing the strengths of specific contacts in each average network with the corresponding contacts in the single networks and *vice versa*, classes B1 and B2 reach the highest degree of relative average similarity between each other (Figure 1B). The specific contacts featured by the single or the average networks of class A are the least dissimilar from those of all other classes, while those of class D1 are the most dissimilar, suggesting that class A GPCRs hold a central position in the structural landscape of the GPCR superfamily. This mirrors deep eukaryotic phylogenetic sequence profiling, which establishes class A as the primary retainer of the ancestral, universally conserved 7TM core sequence framework across early diverging lineages, while class D represents a highly specialized evolutionary branch restricted to fungi.^95,96^

Noticeably, the vast majority of nodes (on average 72%) in class-specific contacts are shared by at least two classes. The percentages of shared nodes go from a minimum of 45.8% for class D1 to a maximum of 80.5% for class B1 (Table S3A and Figure 2). These data suggest that the GPCR superfamily tends to exploit the same 7TM positions to realize class-specific contacts.

The subdivision of GPCRs into classes was captured by principal component analysis (PCA), using the pairwise differences between the average strengths of class-specific contacts as variables, thus supporting the role of such interactions in GPCR classification (Figure S10).

The class A GPCR data set is divided into nine subfamilies, excluding “Orphan” and “Other” GPCRs (Figure S3), while the remaining classes divide in three or one subfamily. Contacts that define specific subfamilies are structurally distinct from those that define broader classes. Specifically, subfamily-specific contacts are less involved in the cytosolic half of the helix-bundle compared to class-specific contacts (Tables S2C and S3B, Figures 3A and S11). In selected subfamilies, the structural portions making the highest relative contributions to these contacts are helices. There is very low overlap in specific contacts between different subfamilies. Only 8.3% (43 out of 515) of these contacts are shared by two or more subfamilies, meaning the vast majority of these structural contacts are entirely unique to their respective subfamily. On average, 45.0% of the nodes involved in these subfamily-specific contacts are conserved. This conservation varies depending on the specific group, ranging from a minimum of 39.8% (Nucleotide subfamily) to a maximum of 67.6% (Amine subfamily), with class A as a whole sitting at 53.1% (Tables S2D and S3B, and Figure S12).

**Figure 3:**
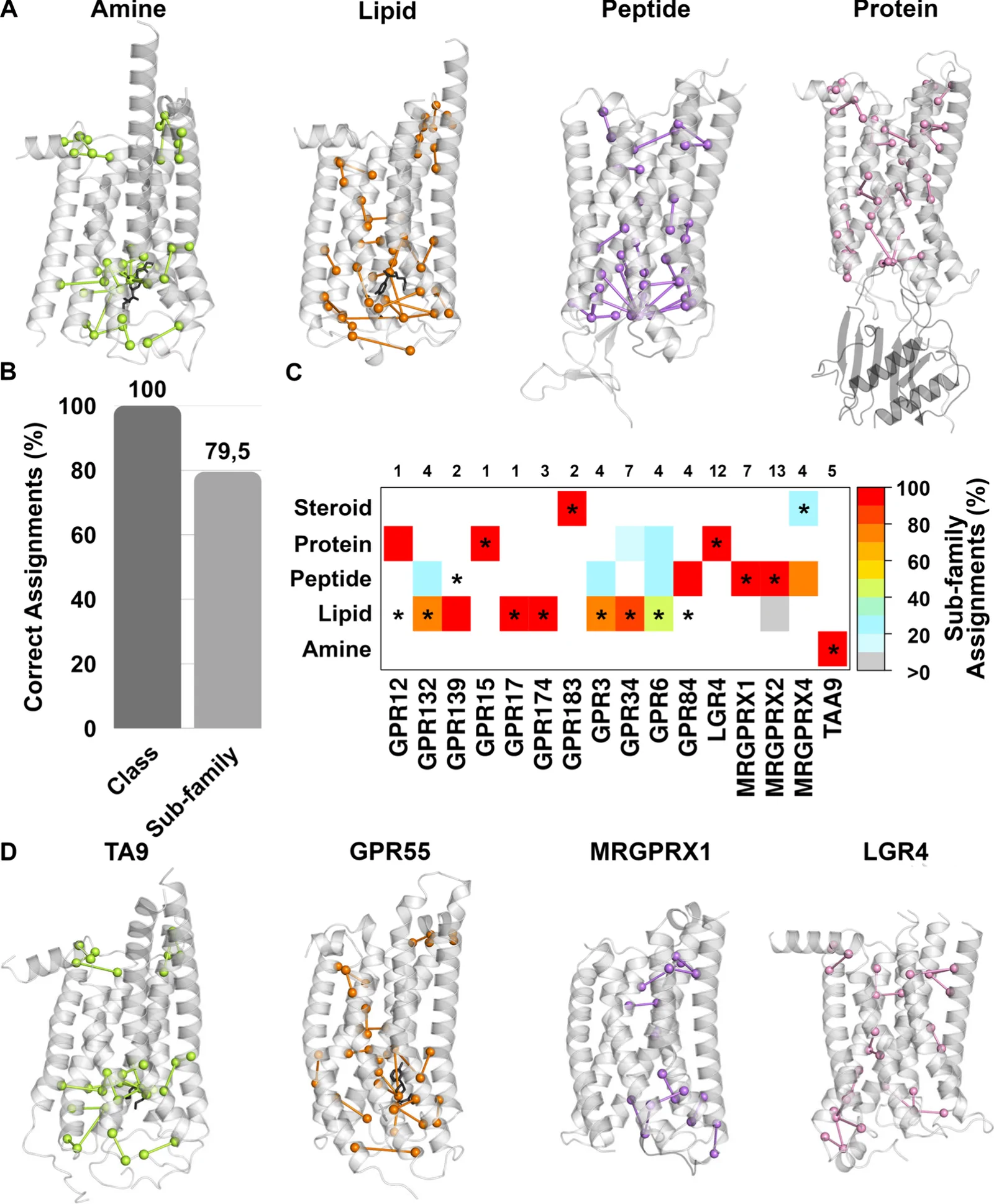
**Assignment of a subfamily to orphan GPCRs.** (A) Cartoon representations of the receptors are shown from a perspective perpendicular to their main axis, with the cytosolic side at the top. Subfamily-specific contacts are mapped into representative structures: amine, 8FYT; lipid, 8GUR; peptide, 9YFY; and protein, 8XX6 subfamilies. (B) The correct assignment statistics of GPCR to the class and subfamily of orphan GPCRs are shown. (C) The matrix shows the assignments (expresses as %) of 74 structures of class A orphan GPCRs to the different subfamilies. The numbers on top of the matrix indicate the number of PDB structures for each Orphan subtype. The asterisk labels the expected subfamily according to the physicochemical features of the bound ligand or data from the literature. (D) The specific contacts in the networks of four orphan GPCRs are shown, which led to the following subfamily assignments: TA9 (PDB: 8IW7), assigned to the amine subfamily; GPR174 (PDB: 7XV3), assigned to the lipid subfamily; MRGPRX1 (PDB: 8DWC), assigned to the peptide subfamily; and LGR4 (PDB: 8XT9), assigned to the protein subfamily. As for the LGR4 structure, the N-terminal domain is not shown.

The model was evaluated on its ability to assign classes and subfamilies to a validation set of 160 orphan GPCR structures of known classes (150 in class A and 10 in class C; see Tables S1 and S5). These structures span 33 subtypes, 14 of which are constitutively active (Tables S1 and S5). For 17 of these subtypes (78 structures total, including GPR12, GPR132, and others listed in Table S5), a ligand-based putative subfamily could be inferred from high-resolution structural complexes and/or functional data^97–113^ to validate predictions. Among these, the 16 subtypes belonging to class A (74 structures) were divided into lipid (8 subtypes, 50%, 28 structures), peptide (3 subtypes, 20%, 22 structures), protein (2 subtypes, 12.5%, 13 structures), steroid (2 subtypes, 12.5%, 6 structures), and amine (1 subtype, 6.25%, 5 structures) putative subfamilies (Table S5).

For the remaining 15 subtypes, a subfamily could not be hypothesized for 6 subtypes (labeled as ‘NA’ in Table S5), whereas a subfamily could only be tentatively hypothesized for 9 subtypes (labeled as ‘Lipid?’ or ‘Peptide?’). Except for GPR30 (60 structures), all of these tentative receptors, including three proton sensors (GPR4, GPR65, and GPR68),^114^ were indirectly associated with the lipid subfamily. Class- and subfamily-specific links were ultimately employed to assign a class to all 160 orphan structures and a subfamily to the 78 orphan structures with an inferred putative subfamily (Table S5).

Remarkably, 100% of the orphan GPCRs could be correctly assigned to a class (Figure 3B). For 11 out of 16 subtypes, at least 75% of the structures were correctly assigned to their expected subfamily. Specifically, all structures belonging to the TAA9 (amine), GPR17 and GPR174 (lipid), MRGPRX1 (peptide), GPR15 and LGR4 (protein), and GPR183 (steroid) subtypes were correctly assigned to their putative subfamilies (Figure 3B). The majority of structures for MRGPRX2 (92.31%), GPR34 (87.7%), GPR132 (75%), and GPR3 (75%) were also correctly assigned to the peptide (MRGPRX2) and lipid (GPR132, GPR3, and GPR34) subfamilies (Table S5 and Figure 3C). In contrast, for four subtypes, GPR12 and GPR84 (lipid), GPR139 (peptide), and MRGPRX4 (steroid), the assignments were incorrect for the majority of available structures. Collectively, 79.5% orphan receptor structures were correctly assigned to a subfamily (Figure 3B-D). Specifically, the percentages of subtypes with the majority of correctly assigned structures are 100% for amine and protein, 66.7% for peptide, 63% for lipid, and 50% for steroid. The number of orphan subtypes is unbalanced toward the lipid subfamily. Noticeably, wrong predictions concerned the exchange of the peptide subfamily with the lipid family, and *vice versa*, consistent with evidence that, phylogenetically, lipid receptors are close to GPCRs promiscuously activated by both lipids and peptides (e.g., FPR2/ALX).^115^

The same approach was employed to reassign the functional state to the 1240 GPCR structures in the trans-class ligand-bound functional-state set. Only 12.6% (13 out of 103) of specific contacts are shared by the OFF and ON states (Table S3C and Figure 4A,B), while the amount of shared nodes is relatively high (i.e., respectively 48.7% and 52.1%) and six of them (1x57, 2x43, 4x53, 6x36, 7x52, and 7x53) are highly conserved. Collectively, more than 50% of functionally state-specific contacts involve conserved nodes (63.7% for the OFF and 50.9% for the ON states).

**Figure 4:**
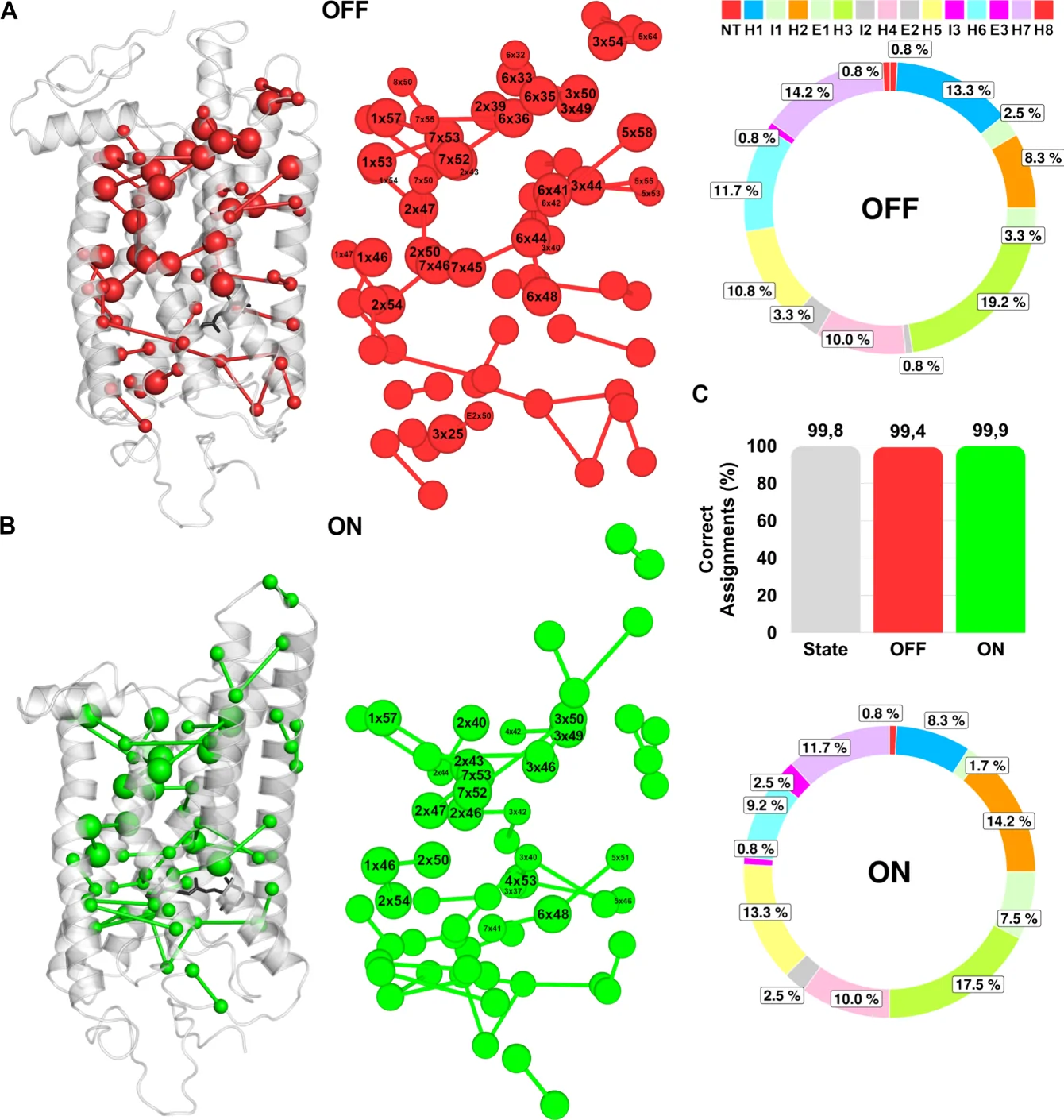
**GPCR functional state-assignment based on specific contacts.** Cartoon representations of the receptors are shown from a perspective perpendicular to their main axis, with the cytosolic side at the top. The specific contacts of the inactive (OFF) (A) and active (ON) (B) states in the GPCR superfamily are mapped onto the structures of dark-state (PDB: 1U19) and Meta II (PDB: 6OYA) rhodopsin. The biggest spheres concern the highly conserved nodes (characterized by average ConSurf conservation grades ≥ 8). Only those nodes and their linked partners are labeled. The contribution of the GPCR portions to the specific contacts of the OFF and ON states is plotted as donut charts. (C) The percentages of correct functional state assignments to the GPCR in the functional-state set are also shown as histograms.

The relative contributions of the different receptor portions to functional state-specific contacts clearly vary between the two states: H1, H6, and H7 contribute more to the OFF state, while H2 and H5 contribute more to the ON state (Table S2E and Figure 4A,B). Remarkably, for both functional states, H3 gives the highest contribution, highlighting the structural and functional centrality of this helix in the GPCR superfamily.

Reassignment of the functional state to the members of the functional-state set was 99.4% and 99.9% correct for the OFF and ON states, respectively (Figure 4C).

Also, functional-state-specific contacts per class allowed an almost perfect assignment of the functional state within classes A, B1, C, and F, for which several structures in both ligand-bound functional states are available. Only for class A OFF and class B1 ON, the percentages of correct assignments are slightly under 100% (i.e., 99.3% and 99.2%, respectively (Figure 5).

**Figure 5:**
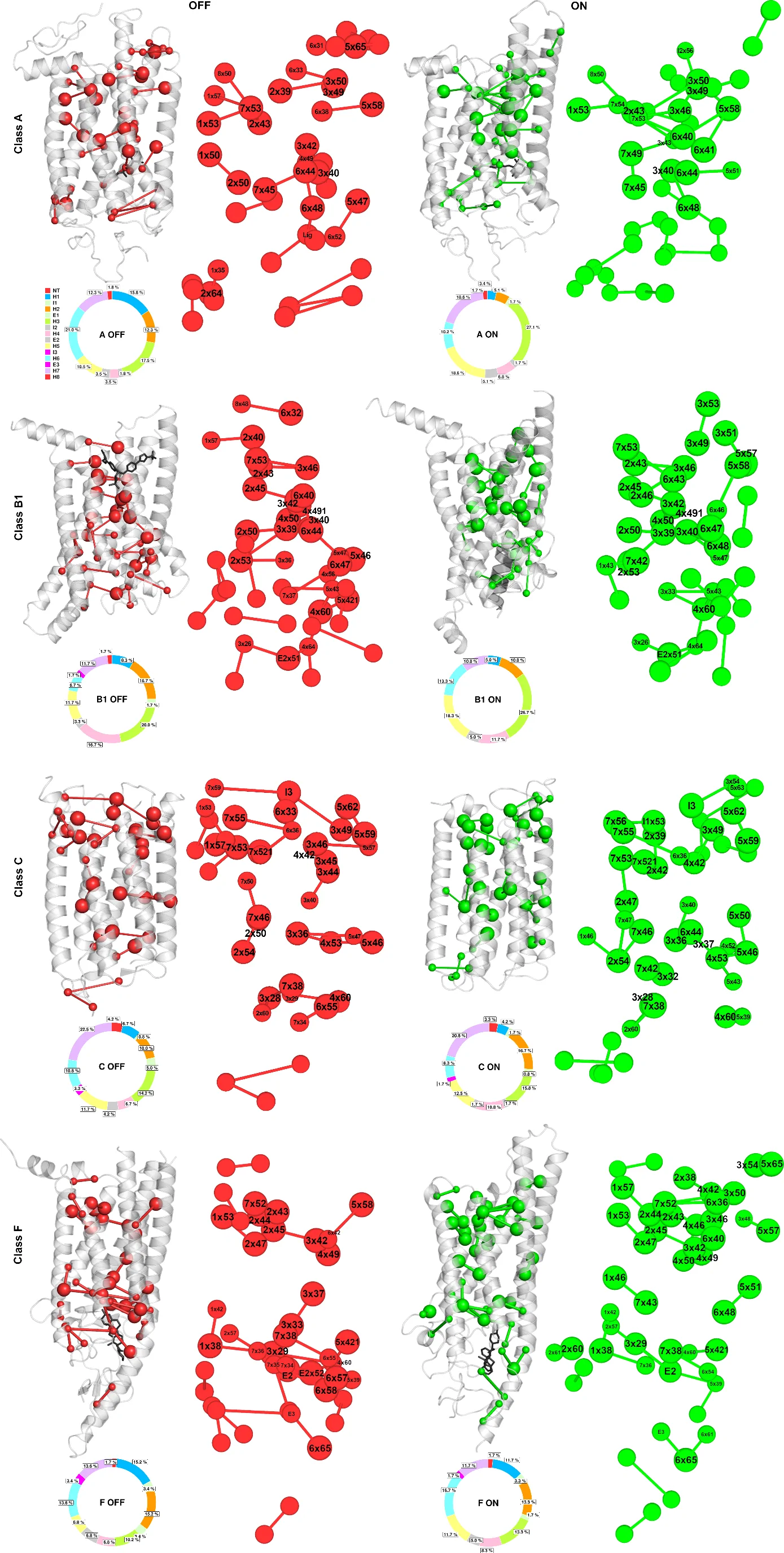
**GPCR functional state-assignment based on specific contacts.** Cartoon representations of the receptors are shown from a perspective perpendicular to their main axis, with the cytosolic side at the top. The contacts specific to the two functional states of classes A, B1, C, and F are shown mapped into selected representatives: class A OFF and ON, 1U19 and 6OYA, respectively; class B1 OFF and ON, 6LN2 and 6X18, respectively (The N-terminus and a portion of the peptide ligand are not shown); class C OFF and ON, 7C7S and 7EB2, respectively (the VFTD and E2 is not shown); and class F OFF and ON, 6D32 and 6XBL, respectively. The biggest spheres concern the highly conserved nodes (characterized by average ConSurf conservation grades ≥ 8). Only those nodes and their linked partners are labeled. For each class-specific functional state, the donut charts of the contribution of the receptor portions to the class functional-state specific contacts are shown.

The structural networks of GPCRs reorganize upon activation, with class A receptors exhibiting the most pronounced conformational divergence between functional states compared to classes B1, C, and F. Upon transitioning from the OFF to the ON state, class A receptors show decreased structural contributions from helices H1, H2, and H6, alongside increased contributions from H3, H4, H5, and H7 (Table S2F and Figure 5). Reflecting these massive rearrangements in the 7TM bundle, class A shares 0% of its specific contacts between the OFF and ON states, whereas classes B1, C, and F retain much higher contact overlaps (46.7%, 32.7%, and 50%, respectively). Despite this complete shift in specific contacts, class A relies on a shared core of ten highly conserved network nodes across both states (1x53, 2x43, 3x40, 3x49, 3x50, 5x58, 6x44, 6x48, 7x45, and 7x53, Table S3D and Figure 5). The other three receptor classes (B1, C, and F) maintain a high fraction of contacts involving highly conserved nodes in both states (i.e., above 70%). This stands in sharp contrast to class A, a difference likely driven by class A’s higher evolutionary variability.

The ability of the specific link-based model to correctly assign the functional state to almost the totality of the 1240 GPCRs in the functional-state set, irrespective of the class, is remarkable and unprecedented, considering the huge and varied numbers of receptor structures.

In a previous study, an activation index, ranging from – 20.0 to 20.7 for the inactive states and from 48.1 to 110.2 for the active states, was inferred as a linear combination of five inter-Cα distances between the following residue pairs 1x53–7x55, 2x50–3x37, 3x42–4x42, 5x66–6x34, and 6x58–7x35.^43^ The training set was based on 16 conformational ensembles of four class A GPCRs (H4 histamine receptor (H4R), β2AR, M2-muscarinic receptor (M2R), and μ-opioid receptor (MOP)) in different functional states.^43^ Validations relied on three test sets of class A GPCRs, one made of 67 ON structures comprising 13 types, 31 intermediate structures comprising 8 types, and 169 OFF structures comprising 25 types. Our model predict correctly the functional state of all the ON and OFF test-set structures shared with our set. None of the five residue pairs contributing to the “activation index” forms functional-state specific links in our model.

Collectively, the GPCR superfamily tends to exploit the same seven-transmembrane positions to achieve class-specific contacts, with the percentage of shared nodes ranging from 45.8% to 80.5%. Across multiple classes, conservation metrics, and functional states (both OFF and ON), H3 consistently provides the highest relative contribution, highlighting its structural and functional centrality. While the overall network relies on a conserved set of nodes, specific contacts heavily rearrange, depending on the class and functional state.

### class A GPCRs: Structural Communication Signatures of the Inactive and Active States

The allosteric communication in class A GPCRs relied on the analysis of the shortest communication pathways. Functional state-dependent allosteric communication was investigated in a nonredundant subset of class A GPCRs (390 structures divided into 145 ligand-bound OFF and 245 ligand- and G protein-bound ON states), i.e., the functional-state-MP set (see Methods and Table S1).

The consensus metapath of the OFF states consists of 67 links, 78% of which involve at least one conserved amino acid (i.e., with an average ConSurf grade ≥ 7; Figure 6A). Indeed, the most conserved residues across all helices except H4 are involved in this metapath. The consensus network and the metapath of the ON state differ significantly depending on whether the G protein is excluded from or included in the calculation (Figure S13). In the absence of the G protein, three communities form on the receptor; the largest community, containing 35 nodes and 44 links, is localized within the orthosteric ligand-binding site (Figure S13A). In contrast, in the presence of the G protein, a single large community forms. This community consists of 54 nodes and 77 links, spanning from the extracellular to the intracellular sides (Figure S13B). Consistently, in the absence of the G protein, the global metapath is more branched, comprising 62 nodes and 63 links; whereas in the presence of the G protein, the receptor portion of the metapath consists of only 14 nodes and 14 links (Figures 6B and S13 C,D). The most intracellular node of the metapath is the highly conserved 3x50, the arginine of the E/DRY motif, which is engaged in an intermolecular link with G.H5.25 of the G protein. Thus, the presence of the G protein reshapes the structural network of the receptor by promoting the interconnection of the two poles of the helix bundle and prioritizing a few select communication pathways from the orthosteric binding site of the receptor to the G domain of the G protein.

**Figure 6:**
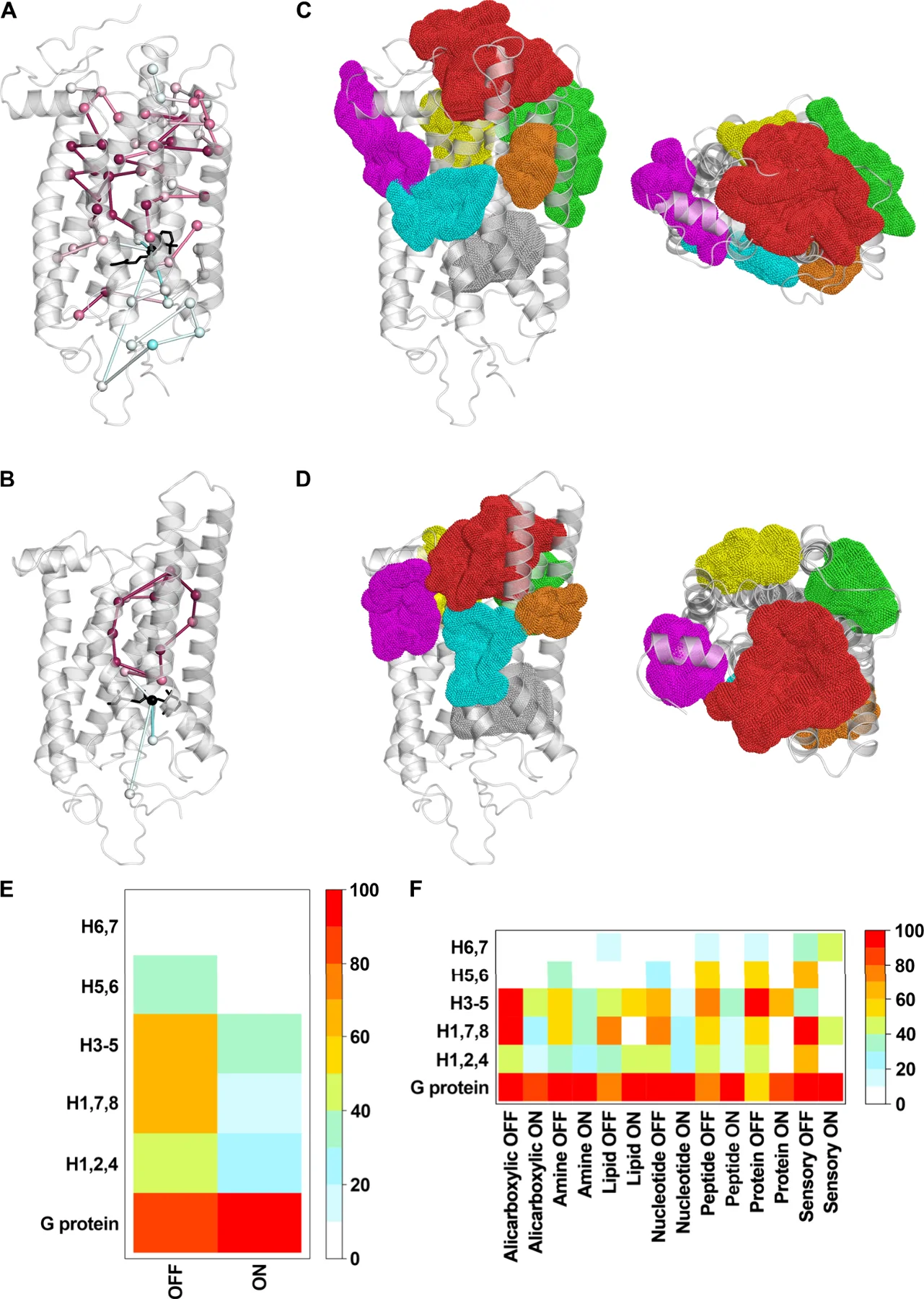
**Consensus metapaths and prediction of allosteric cavities.** The consensus metapaths of class A GPCRs computed on the metapath-set in the OFF (A) and ON (B) states are shown, mapped on the structures of rhodopsin in the OFF (1U19) and ON (6OYA) states. Nodes are colored according to the ConSurf conservation grades, whereas links are colored according to the average ConSurf grades of the connected nodes. Cartoon representations of the receptors are shown from a perspective perpendicular to their main axis, with the cytosolic side at the top. (C and D) Two views of the 1U19 (C) and 6OYA (D) structures are shown with the predicted cavities mapped on them. Cavities are colored according to their location: orthosteric site (gray), G protein binding site (G protein, red), external faces of H1, H2, and H4 (H1,2,4, yellow), H1, H7, and H8 (H1,7,8, magenta), H3, H4, and H5 and interconnecting loop (H3–5, green), H5 and H6 (H5,6, orange), and H6 and H7 (H6,7, cyan). Only for 6OYA, the shown cavity H1,2,4 is the one predicted on the 7CKX structure. (E and F.) Percentages of structures whose metapath touches a given cavity with at least one node. For this calculation, each cavity is contributed by those amino acid residues, which participate in the considered cavity in at least 70% of the 145 ON and 245 ON structures. In E, the structures are divided according to their functional state (OFF/ON), while in F the structures are divided according to functional state and subfamily.

In parallel to PSN-based metapath calculation, potential allosteric sites in class A GPCRs were predicted on the whole metapath set by the updated version of the CavityPlus Web server.^89^ The six allosteric cavities predicted by the server as correlated with the orthosteric binding site are essentially located in the cytosolic half and are the same in both functional states, differing only in their extension and shape (Figure 4C).

The largest and unique core-facing allosteric cavity is the G protein-binding site (red), whereas the remaining five cavities are located on the external faces of a) H1, H2, and H4 (H1,2,4, yellow); b) H1 and H7 (H1,7, magenta); c) H3, H4, and H5 (H3–5, green); d) H5 and H6 (H5,6, orange); and e) H6 and H7 (H6,7, cyan) (Figure 4C). Independent of the functional state, in almost 100% of structures, the metapaths touch the G protein cavity with at least one node (Figure 6D). In contrast, the involvement of metapath nodes in the other allosteric cavities, with emphasis on H1,2,4, H1,7, and H3–5, is more frequent in the OFF states, in line with the extended and branched metapath in the absence of the G protein. The trend described above is confirmed at the subfamily level, which puts emphasis also on the H5,6 cavity for the OFF states of peptide, protein, and sensory GPCRs (Figure 6D). Matching metapath nodes with the most recurrent cavity residues (i.e., contributed by at least 70% of the structures in the OFF state) suggests that those cavities are the target by small allosteric modulators of different class A GPCRs.

Prediction of the highly druggable G protein binding site as an allosteric cavity not only for the ON states, deputed to recognizing the G protein, but also for the OFF states is in line with it being the target of allosteric antagonists and inverse agonists (Figure S14). Indeed, several allosteric antagonist drug candidates for chemokine receptors 2, 7, and 9 and an inverse agonist of GPR61 bind to the G protein cavity by blocking activation-associated conformational changes and formation of the G-protein-binding interface.^116–120^

Collectively, the allosteric cavities other than the G protein binding site predicted in this study proved to be targets of several negative allosteric modulator (NAM) and positive allosteric modulator (PAM) compounds. In this respect, the H3–5 cavity is the target of both NAMs of C5aR^121^ and β2AR^122^ and PAMs of D1 dopamine receptor (D1R),^123^ free fatty acid 1 and 2 receptors (FFA1R and FFA2R, respectively),^124,125^ MOP,^126^ GPR4,^127^ and GPR99.^128^ Remarkably, such a cavity is prone to bind molecules differing not only in their function, like PAMs and NAMs, but also in their physicochemical character, like polar and hydrophobic compounds. The latter include docosahexanoic Acid (DHA), lysophosphatidyl choline (LPC), and cysteinyl leukotriene E4 (LTE 4) bound to FFA1R, GPR4, and GPR99, respectively. Similar to cavity H3–5 though by a lesser extent, cavity H5,6 is recognized by both NAM and PAM ligands of the CXCR3 chemokine receptor^129^ and GPR88,^130^ respectively. Finally. the cavity H1,2,4, overlapping with a conserved site of cholesterol interaction,^131^ has been found bound to a NAM^132^ compound in the CB1R, whereas the cavities H6,7 and H1,7,8 have been found bound to PAM compounds in the FFA2R^125,133^ and GPR84,^134^ respectively (Figure S14).

The marked difference in the structural communication of OFF and ON states was also captured by the PCA using as variables the strengths of links in the metapaths computed on the 390 structures in the considered set (Figure 7A-C). Indeed, PC1 effectively separated the two states (Figure 7B,C). Fully correct assignments of test-set structures to the two functional states were achieved on the basis of the distances between the principal coordinate of each test-set structure and the centroids of the OFF and ON subsets (Figure 7C).

**Figure 7:**
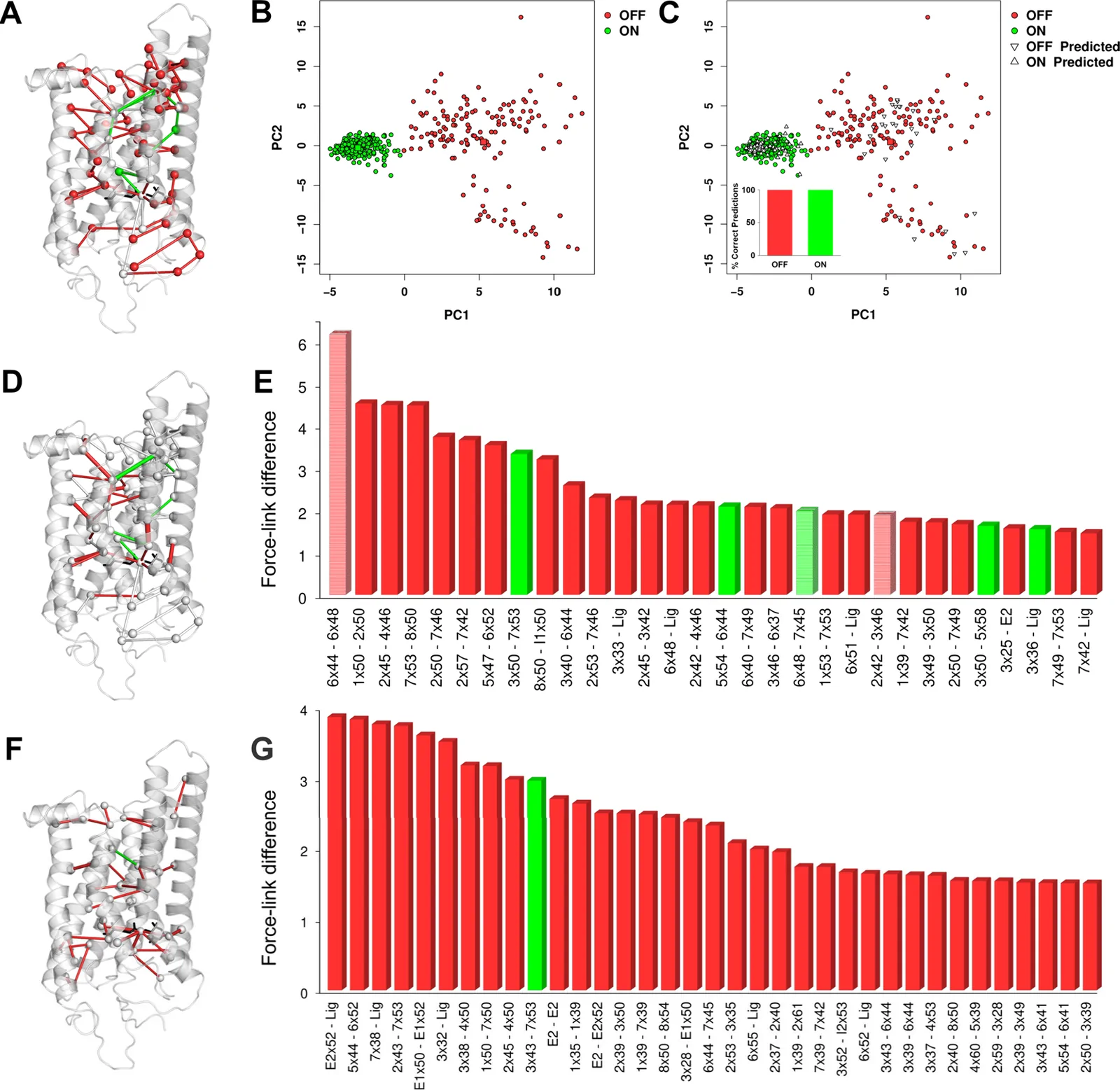
**Functional state-dependent structural communication.** (A) The difference between the metapaths of the OFF and ON states is shown. Red and green links are, respectively, specific to the OFF and ON states, whereas white links are shared by the two states. (B and C) Scatter plots of the first two principal components (PC1 and PC2), using the strength of the metapath-links as variables. The bigger squares indicate the centroids of the two states. Tip-down and tip-up white triangles in C concern the test-set structures predicted as OFF and ON, respectively. The percentages of correct predictions are shown in the inset histograms. (D and E) 3D representation and plot of those links that are specific for the OFF and ON states in the consensus metapaths and also hold an average difference (between the two states) in contact-strength and variance in PCA above a cutoff (i.e., ≥ 1.5 and ≥ 0.5, respectively). The color of the histograms corresponds to the signaling state, in which the link prevails in strength. The difference in link-strength is reported on the ordinate. Horizontal white lines in some histograms mean that the considered link is shared by the two states in the consensus metapath, while it prevails in one of the two states in the PCA. (F and G) 3D representation and plot, respectively, of those links that do not appear in the consensus metapaths and hold the average difference in link-strength and variance in PCA above the cutoff in either one of the two functional states. Cartoon representations of the receptors are shown from a perspective perpendicular to their main axis, with the cytosolic side at the top.

The links that are peculiar to each of the two states in the consensus metapaths (i.e., colored red and green in Figure 7A) also contribute to separating the two states in the PCA and are essentially located in the intracellular half. Specifically, the contacts that contribute the most to distinguish the OFF state involve, among others, the highly conserved amino acid residues 1x50, 2x50, 3x49, 3x50, and 3x51 (the last three belonging to the E/DRY motif), 6x44, 6x48, 7x49, and 7x53 (both of the NPxxY motif), and 8x50. The intrahelix contact with the highest difference in PCA variance in favor of the OFF state, 6x44–6x48, is shared by the metapaths of the two states (Figure 7D,E), thus highlighting the universal role of the two residues in the structural communication of class A GPCRs. Peculiar contacts of the ON states include those that involve the arginine of the E/DRY motif and both the highly conserved tyrosines at positions 5x58 and 7x53, which fall among the five contacts that exhibit the highest variance difference in favor of the ON states, i.e., 3x50–5x58, 3x50–5x53, 5x53–6x44, and 6x48–7x45. Noticeably, the 6x48–7x45 contact is shared by the metapaths of the two states (Figure 7D,E). Additional high-variance contacts in PCA, not contributing to the two consensus metapaths, form two separate groups in the two halves of the helix bundle (Figure 7F,G). Seven out of the 16 contacts in the extracellular half involve the ligand, whereas the contacts in the cytosolic half involve highly conserved amino acid residues such as 2x50, 3x49, 3x50, 4x50, 6x44, 7x45, 7x53, and 8x50 (Figure 7F,G).

In summary, the synergic combination of consensus metapath comparisons and PCA, using the strength of links as variables, could infer those nodes that are central in the functional-state-independent and -dependent structural communication in class A GPCRs.

### Class A GPCRs: Structural Communication Signatures of the Gi/o and Gs Signaling States

Signaling-state-dependent allosteric communication was investigated in a nonredundant subset of 202 ligand- and G-protein-bound class A GPCRs, i.e., signaling set, which was split into Gi/o (151 structures) and Gs (51 structures) sets, thus allowing comparison of the metapaths computed on the two average networks.

The highly conserved G protein α-subunits share a common mode of class A GPCR recognition characterized by the docking of the C-terminal helix of the G domain (G.H5) into a receptor cavity participated by H2, H3, H5, H6, H8, and I2 as well as the interaction of the N-terminal helix (G.HN), the interswitch loop (i.e., G.s2s3), the G.h4s6 loop, and G.S6 with I2, I3, H5, and H6 of the receptor. Yet, the specific docking modes of the different G protein types, Gi/o and Gs in this study, differ in the orientation of G.HN and G.H5 (Figure 8A). In detail, the inclination of G.H5 relative to the receptor core is higher for Gs than Gi/o, thus resulting in a tighter interface in the complexes involving Gs (Figures 8A, S15, and S16). These differences in the architecture of the receptor–G protein interface reflected on signal transfer between the two proteins. It is worth recalling that the αβα two-layer sandwich architecture of the G domain consists of a central parallel β-sheet sandwiched between two helix-layers (sheet-helix interfaces 1 and 2; Figures S5, S15, and S16). As for interface-1, G.H1 packs against G.S2 and G.S4, while G.H5 packs against all strands but G.S4; as for interface-2, G.H2 packs against G.S1, G.H3 packs against G.S4, and G.H4 packs against G.S5 and G.S6. Thus, G.H5, central in receptor recognition, is wrapped by the parallel β-sheet.

**Figure 8:**
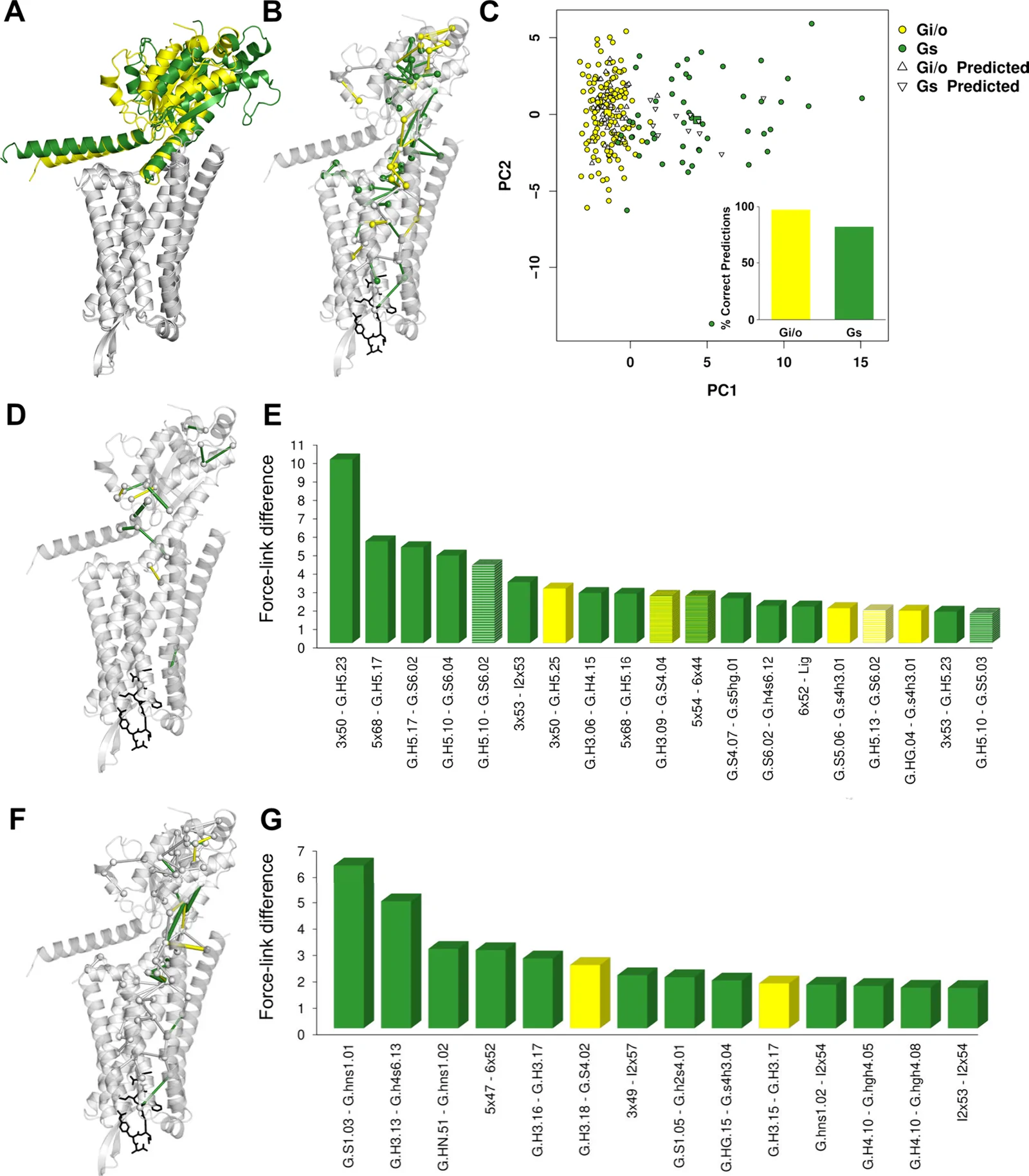
**Signaling state-dependent structural communication.** (A) The overlapped complexes between the CCKA receptor and Gi/o (yellow, PDB: 7EZH) and Gs (green-forest, PDB: 7MBX) are shown. (B) The difference between the metapaths of the Gi/o and Gs signaling states is shown. Yellow and dark-green links are, respectively, specific to the Gi/o and Gs states, whereas white links are shared by the two states. **C**. Scatter plot of the first two principal components (PC1 and PC2), by using the strength of the metapath-links as variables. The bigger squares indicate the centroids of the two signaling states. Tip-up and tip-down white triangles concern the test-set structures predicted as Gi/o- and Gs-coupled, respectively. The percentages of correct predictions are shown in the inset histograms. (D and E) 3D representation and plot of those links that are specific for the Gi/o and Gs states in the consensus metapaths and also hold an average difference in link-strength (between the two states) and a variance in PCA above a cutoff (i.e., ≥ 1.5 and ≥ 0.5, respectively). The color of the histograms corresponds to the signaling state, in which the link prevails in strength. The difference in link-strength is reported on the ordinate. Horizontal white lines in some histograms mean that the considered link is shared by the two states in the consensus metapath, while it prevails in one of the two states in the PCA. Horizontal dark-green lines on yellow histograms and, *vice versa*, horizontal yellow lines on dark-green histogram mean specific for Gs in the consensus metapath but prevailing in Gi/o in the PCA, and *vice versa*. (F and G) 3D representation and plot of those links that do not appear in the consensus metapaths and hold an average difference in link-strength and variance in PCA above the cutoff in either one of the two signaling states. Cartoon representations of the receptors are shown from a perspective perpendicular to their main axis with the cytosolic side at the top.

On the G domain, the Gi/o metapath involves the interface-1 with contacts between G.H5 and both G.S6 and G.S5, and the interface-2 with contacts between G.H3 and G.S4. The passage from one interface to the other is mediated by contacts between G.HG and G.S5. The metapath thus ends on G.H2, the switch II (swII) region at the interface with the β-subunit (Figure S15).

The Gs metapath involves all the elements of the αβα-sandwich, making links at both interface-1 (between G.H5 and both G.S6 and G.S5, and between G.H1 and the G.s1h1 loop) and interface-2 (between G.S4 and both G.H3 and G.H4). Similar to the Gi/o metapath, the Gs metapath concludes at G.H2. The Gi/o metapath differs fundamentally from the Gs metapath in how the receptor nodes 5x58 and 7x53 communicate with the more extracellular nodes and in its contacts at both the receptor–G protein interface and within the G domain.

While for the Gi/o metapath, only two nodes on the receptor (3x50 and, with low recurrence, 6x32) are linked to G.H5, for the Gs metapath a number of intermolecular links between G.H5 and the receptor I2, H3, and H5 are formed. As a consequence, differently from the Gi/o metapath, the Gs metapath involves all the secondary structure elements of the αβα-sandwich (Figures 8B, S15, and S16). In spite of clear divergences, the two metapaths also share, among others, nodes on G.H5, G.S6, G.S5, G.S4, G.H3, and G.H2 (Figure 8B).

The differences between the two consensus metapaths were captured by the PCA performed on all structures in the Gi/o and Gs subsets, employing the strength of metapath links as variables. The distance between the principal coordinate of each test-set structure and the centroids of the two signaling subsets allowed 97.3% and 90.9% correct assignments of the test-set complexes to the Gi/o and Gs signaling, respectively (Figure 8C).

Some of the links singular to each of the two signaling states in the consensus metapaths (i.e., colored yellow and dark green in Figure 8D,E) also contribute to separate the two states in the PCA (Figure 8D,E). The list included, among others, the links between a) 3x50 and G.H5.23 or G.H5.25 for Gs and Gi/o, respectively; b) H5 and G.H5 for Gs; c) G.H5 and G.S6 for Gs; d) H3 and I2 for Gs; and e) G.HG and G.s4h3 loop for Gi/o. Almost all of those links are located at the receptor-G protein interface and on the G domain (Figure 8D,E). Some additional interactions that contributed to signaling-state separation only in PCA are peculiar to Gs signaling. These involve, but are not limited to, G.HN, G.S1 and the interconnecting loop, G.H3, G.H4, G.hgh4, and G.h4s4 loops, and I2 (Figure 8F,G).

Collectively, the integration of metapath-link comparison and PCA highlighted the specificities in the allosteric communication associated with Gi/o and Gs signaling.

## Discussion

Structure network analysis represents an invaluable tool for deciphering GPCR function that strongly relies on the communication between the extracellular and intracellular poles of the 7TM helix bundle. GPCRs are very dynamic proteins, which exist as conformational ensembles also dictated by the type of bound ligand and/or intracellular proteins.^1,2,135^ Functional dynamics of GPCRs can now be inferred from the huge number of atomic-level-resolution structures released in the PDB.

Translation of biomolecular systems into structure graphs, condensation of the major communication pathways into metapaths incorporating systems’ dynamics even in single structures, computation of consensus networks and consensus metapaths, and their comparisons are all unique features of our method implemented in the standalone software PSNtools^74^ and available via the webPSN server.^60,71^ This method was exploited here to explore the landscape of GPCR states spanned by the PDB structures through their noncovalent intramolecular and intermolecular contacts. Once translated into structure graphs, the 1568 nonorphan GPCRs in the data set were variedly assembled in average networks and, at a given hierarchy level, they were compared based on the strength of the inter-residue contacts either in the whole networks or in the communication metapaths. The analysis of more than 2-million contacts extracted from the GPCR structure-graphs revealed that 30 specific contacts are enough to encode the signatures of receptor aggregations in different classes, subfamilies, types, subtypes, and functional states. The strengths of such specific contacts worked out to properly reassign each GPCR structure to a given aggregation.

To infer GPCR activation mechanisms, previous structural network analyses relied on binary contact maps, defining a residue contact if the atomic distance fell below the sum of their van der Waals radii plus 0.5 Å.^56,63,73^ To eliminate local noise, backbone contacts between residues fewer than four amino acids apart in the primary sequence were excluded. Alternatively, Zhou and co-workers used a higher-resolution approach based on the residue–residue contact score (RRCS). For any residue pair, the RRCS sums a piecewise (plateau-linear-plateau) atomic contact score across all heavy atom pairs, restricted to adjacent residues within four amino acids in the sequence.^70^ Two of those studies inferred common class A GPCR activation pathways using remarkably limited data sets. The first comprised only 27 structures (21 inactive and six active states).^63^ The second evaluated six different GPCRs in both inactive and active states for an initial identification of conserved residue contact rearrangements; this was followed by a “family-wide” conservation analysis of residue contact patterns using 142 inactive structures (from 38 receptors) and 27 active structures (from eight receptors).^70^ Among these, active-state structures bound to the G domain of a G protein totaled one in the first analysis and seven in the second. Relying on such a low informative sets, activation pathways were proposed based on comparison of residue contacts between the two states but without employing any algorithm for shortest pathway predictions.^63,70^ In a more recent study analyzing 521 structures, activation microswitches across Classes A, B1, C, and F GPCRs were inferred by comparing residue-contact frequencies across inactive, active, and intermediate states. The thresholds for frequency differences were class-dependent, scaled according to the structural coverage within each GPCR class. Of the 177 structures annotated as active, only 80 were in complex with at least the G domain of the G protein.^73^ Regarding class A, only 6 out of the 24 highly recurrent contacts in the OFF states (1x53–7x53, 2x39–3x50, 2x43–7x53, 3x49–3x50, 5x58–6x38, and 7x53–8x50) and 6 out of 8 highly recurrent contacts in the ON states (3x40–6x48, 3x46–7x53, 3x50–5x58, 5x51–6x44, and 5x58–6x40) identified in that work overlap with the 30 specific contacts determined for the respective functional state in our current investigation.^73^ These shared contacts primarily involve the intracellular half of the receptor. In contrast, only a single link in the OFF state of Classes B1 and F was shared between both studies, and no common contacts were identified for class C. These discrepancies underscore that the findings of previous studies are not easily comparable to ours for several reasons. First, our approach computes contacts exclusively from side-chain heavy atoms (excluding glycines). Second, it employs an automatically calculated interaction strength cutoff to filter meaningful noncovalent interactions from otherwise noisy, high-density contact data. This interaction strength forms the foundation for identifying specific contacts that dictate GPCR aggregation in classes, families, subfamilies, types and functional states, a unique feature of this study. Furthermore, the structural communication in our model was inferred using algorithmic predictions of the shortest communication pathways. The latter, filtered according to the cross-correlations of atomic motions, ultimately converged into global metapaths that summarize the communication networks within the inactive and active states of class A GPCRs. Notably, the strength of these metapath links successfully differentiated inactive from active conformations, as well as Gi and Gs signaling states based on PCA models. For the active states, we strictly selected GPCR complexes containing at least the G domain of the G protein; these represent the fully active states, and our data demonstrate that the presence of the G protein significantly modulates the structural network. Consistently, all physiologically relevant interacting partners were explicitly incorporated into the network calculations. Furthermore, the scale of this expanded data set is unprecedented and substantially outperforms previous studies. Ultimately, the robustness of this approach is demonstrated by the high classification accuracy across multiple structural levels: class (100%), subfamily (99.7%), type (99.6%), subtype (94.4%), and class-independent inactive (99.4%) and active (99.9%) states. This specific-contact-based model was further leveraged to assign the class and subfamily for 160 orphan GPCRs. Moreover, PCA models utilizing metapath link-strengths correctly predicted the specific signaling state (Gi versus Gs) for 97.3% of receptor-Gi complexes and 90.9% of receptor-Gs complexes.

The GPCR superfamily primarily utilizes the same 7TM positions to form specific contacts, with helices H3, H5, H6, and H7 providing high and/or highly variable relative contributions across most structural clusters. This pattern stems from the fact that, in most classes, the cytosolic half of H3 is packed inward, making contact with all helices except H1, whereas H5, H6, and H7 undergo distinct conformational and orientational shifts depending on the specific class and functional state. Incidentally, H3 was defined as a hub in the consensus network of interhelix contacts inferred from the analysis of a very small set (20 structures belonging to five subfamilies and 11 types) of class A GPCRs.^56^

Moving hierarchically from classes to subtypes, the structural contributions of the receptor halves shift progressively toward the extracellular regions, involving the orthosteric ligand binding site. Within this framework, subfamily-specific contacts were leveraged to assign classifications to 33 subtypes of orphan GPCRs, spanning 160 structures. Notably, class assignments were correct for all of the evaluated structures. Regarding class A orphans, subfamily assignment was validated for 16 subtypes using putative subfamilies inferred from high-resolution structural complexes and/or functional data,^97–113^ yielding a 79.5% accuracy rate. Intriguingly, incorrect predictions almost exclusively involved misclassification between the lipid and peptide subfamilies. This finding is consistent with phylogenetic evidence indicating that lipid receptors are closely related to GPCRs promiscuously activated by both lipids and peptides (e.g., FPR2 and ALX).^115^

Recent GPCR deorphanization strategies have relied on reverse pharmacology integrated with high-throughput signaling detection technologies, capitalizing on the growing availability of genomic, transcriptomic, metabolomic, and peptidomic data.^24–28^ The primary objective of these highly demanding approaches is to pair cognate ligands with the corresponding orphan receptors. Here, we challenged our specific link-based GPCR classification method to assign subfamilies to orphan receptors without relying on ligand pairing. Independent of validation, the model assigns the vast majority (84%) of the 33 orphan subtypes to lipid, peptide, and protein subfamilies. In particular, the bias toward the lipid subfamily may stem from the high structural plasticity of lipid GPCR binding sites, as captured by cryo-EM structures.^115^ Ultimately, this network model aligns with the established consensus that the majority of orphan GPCRs cluster into lipid and peptide subfamilies due to evolutionary biology, ligand biochemistry, and historical limitations in pharmacology.^23^

Upon activation, class A GPCRs undergo the most radical structural network reorganization of all classes, retaining 0% of their state-specific contacts when transitioning from the OFF to the ON state (compared to 32–50% for Classes B1, C, and F). Despite this complete contact turnover across the 7TM bundle, class A activation remains anchored by a highly conserved core of ten universal network nodes (namely 1x53, 2x43, 3x40, 3x49, 3x50, 5 x58, 6x44, 6x48, 7x45, and 7x53), which are all fundamental for the process of GPCR activation,.^51,70,135,136,141,144,145,147,149^ Collectively, within the class A functional-state-specific networks, 24 out of 46 nodes (52%) in the OFF state and 22 out of 41 nodes (54%) in the ON state are evolutionarily conserved (exhibiting average ConSurf conservation grades ≥ 7). Among these conserved nodes, 96% in the OFF state and 86% in the ON state are known to be functionally relevant for GPCR activity.^18,51,70,136–159^

The ten highly conserved nodes shared by the specific contact networks of class A OFF and ON states serve as key components in the consensus communication pathways that distinguish the inactive and active conformations. Indeed, analyzing the most representative shortest communication pathways in the form of metapaths revealed that, irrespective of the functional state, GPCRs rely on highly conserved nodes to mediate structural communication between the orthosteric ligand binding and G-protein binding sites. These communication metapaths also intersect with several sites known to accommodate allosteric antagonists, NAMs, and PAMs in high-resolution structures.^116–130,132–134^ Crucially, these same sites were predicted to be in allosteric communication with the orthosteric pocket by a parallel, independent approach using CavityPlus.^89^ Validation from cryoEM structural complexes^116–130,132–134^ confirms that combining metapaths with CavityPlus predictions effectively identifies druggable allosteric sites in GPCRs.

The structural communication divergence between the OFF and ON states is further amplified by the presence of the G protein, which channels structural communication to a few specialized pathways. These pathways involve the aromatic cluster in H6 (6x44 and 6x48), polar residues in H7 (7x45 and 7x49), two cytosolic tyrosines in H5 (5x58) and H7 (7x53), and the E/DRY arginine in H3 (3x50), which serves as a common recognition point for the G protein. These findings suggest that in class A GPCRs, G-protein binding reorganizes the receptor’s structural network, funneling the multitude of potential shortest communication pathways into a few preferential routes that culminate at the fully conserved arginine, a key mediator of G-protein recognition.This crucial insight, derived from computing the meta-communication pathways in both the presence and absence of the G protein, is conceptually consistent with previous network analyses suggesting the convergence of activation pathways within the G-protein-coupling region.^63^

The receptor-G protein interface is tighter in Gs than in Gi/o, featuring a higher number of intermolecular contacts that participate in the consensus metapath. This suggests a less dynamic interface and a more intense, faster GPCR-Gs structural communication compared with the GPCR-Gi system. Consistently, despite sharing several common contacts, communication within the G domains of Gi/o and Gs diverges, which may have implications for the dynamics of G-protein activation and interaction with effector and regulatory proteins.

In summary, this extensive structural network analysis of GPCRs suggests that the determinants of their classification and differentiation, by sequence, functional state, and signaling state, reside within their structural communication. GPCRs leverage the same overall fold and even conserved amino acids to form specific contacts specialized for functional diversification. The G protein acts to unify structural communication within the 7TM bundle, generating signaling divergent pathways that extend from the receptor interface to the G domain.

Moving forward, the specific link-based computational models developed in this study will be expanded to integrate the rapidly growing number of GPCR structures in PDB.

The single structure networks analyzed here are stored in the continuously updated psnGPCRdb,^75^ which serves as a valuable resource for unraveling GPCR function with critical implications in drug design.

## Supplementary Material













## Notes

PDB codes and annotations concerning the GPCR set (XLSX)
Distributions of nodes in specific contacts according to conservation or position (XLSX)
Lists of specific contacts (XLSX)
Average conservation grades (XLSX)
Assignments of class and family to 160 orphan GPCRs (XLSX)
Supporting figures (PDF)
