# Supplementary material for: Landscape of G Protein-Coupled Receptor Function from Structure Networks
Source: JACS Au. 2026 Jul 30;6(8):4718–39. doi: 10.1021/jacsau.6c00851 (PMC13508081; doi:10.1021/jacsau.6c00851)
Supplement: Supplementary file 1 [file au6c00851_si_006.pdf]

Landscape of G protein-coupled receptor function from structure networks

Sara Gentile,[1‡] *Angelo Felline,*[1‡] *Sara Mazzali*[1] *and Francesca Fanelli,*[1*]

[1]Department of Life Sciences, University of Modena and Reggio Emilia, via Campi 103, 41125, Modena, Italia.

*Corresponding author, fanelli@unimo.it
‡Equally contributing authors

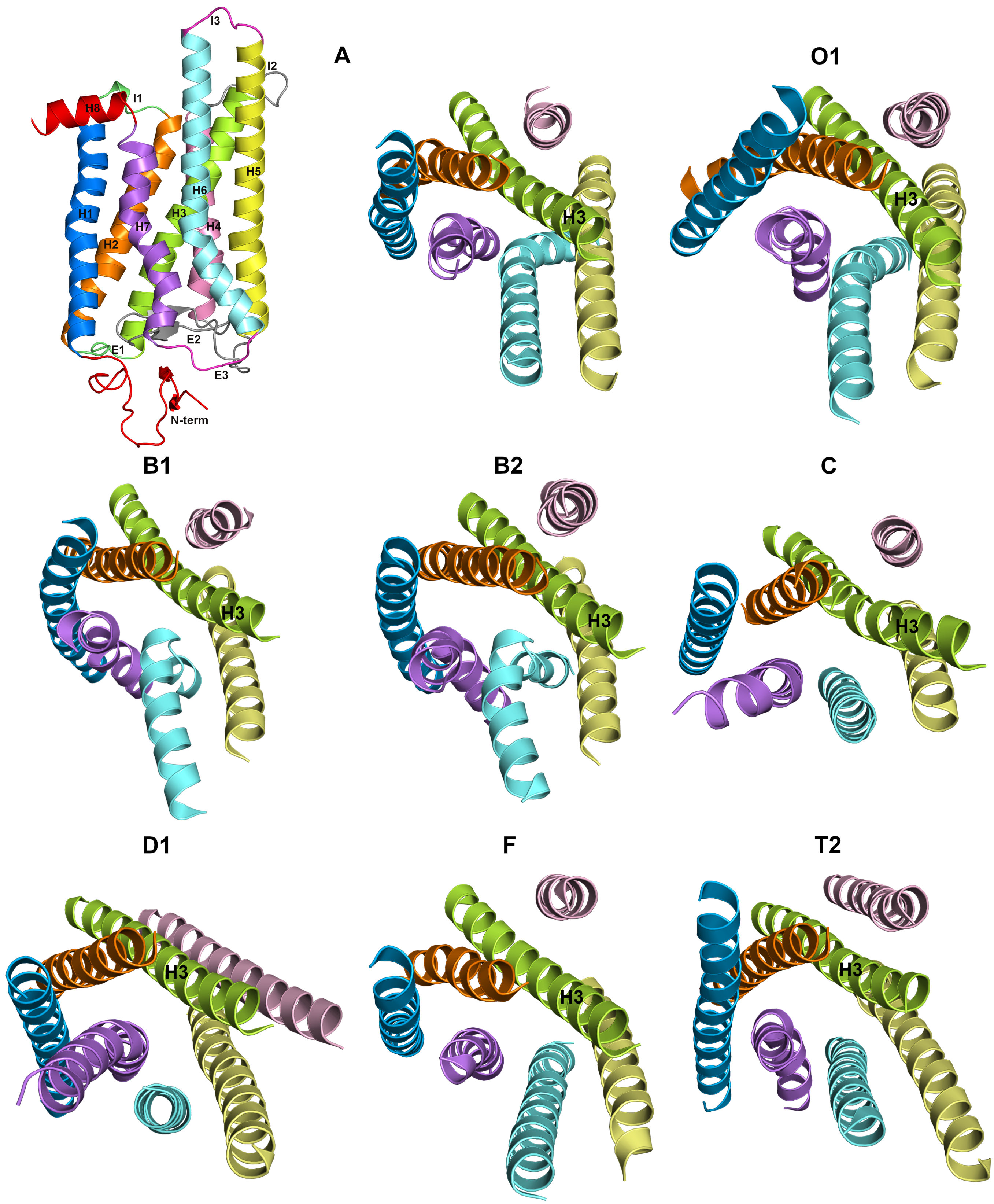

Figure S1. Crystal structure of rod opsin. Cartoon representation, in a direction perpendicular to the receptor main axis, of rod opsin structure as a representative of class A. The intracellular side is at the top. H1, H2, H3, H4, H5, H6, and H7 are respectively colored blue, orange, lemon-green, pink, yellow, cyan, and violet; the N- and C-termini, including H8, are red, I1 and E1 are green-lime, I2 and E2 are gray, and I3 and E3 are magenta. The seven helices of representative structures of all classes (class A, 6OYA; B1, 6WZG; B2, 7WU5; C, 7EB2; D1, 7AD3; F, 6OT0; O1, 8F76; and T2, 7XP6) are shown as well seen from the intracellular side in a direction parallel to the receptor main axis.

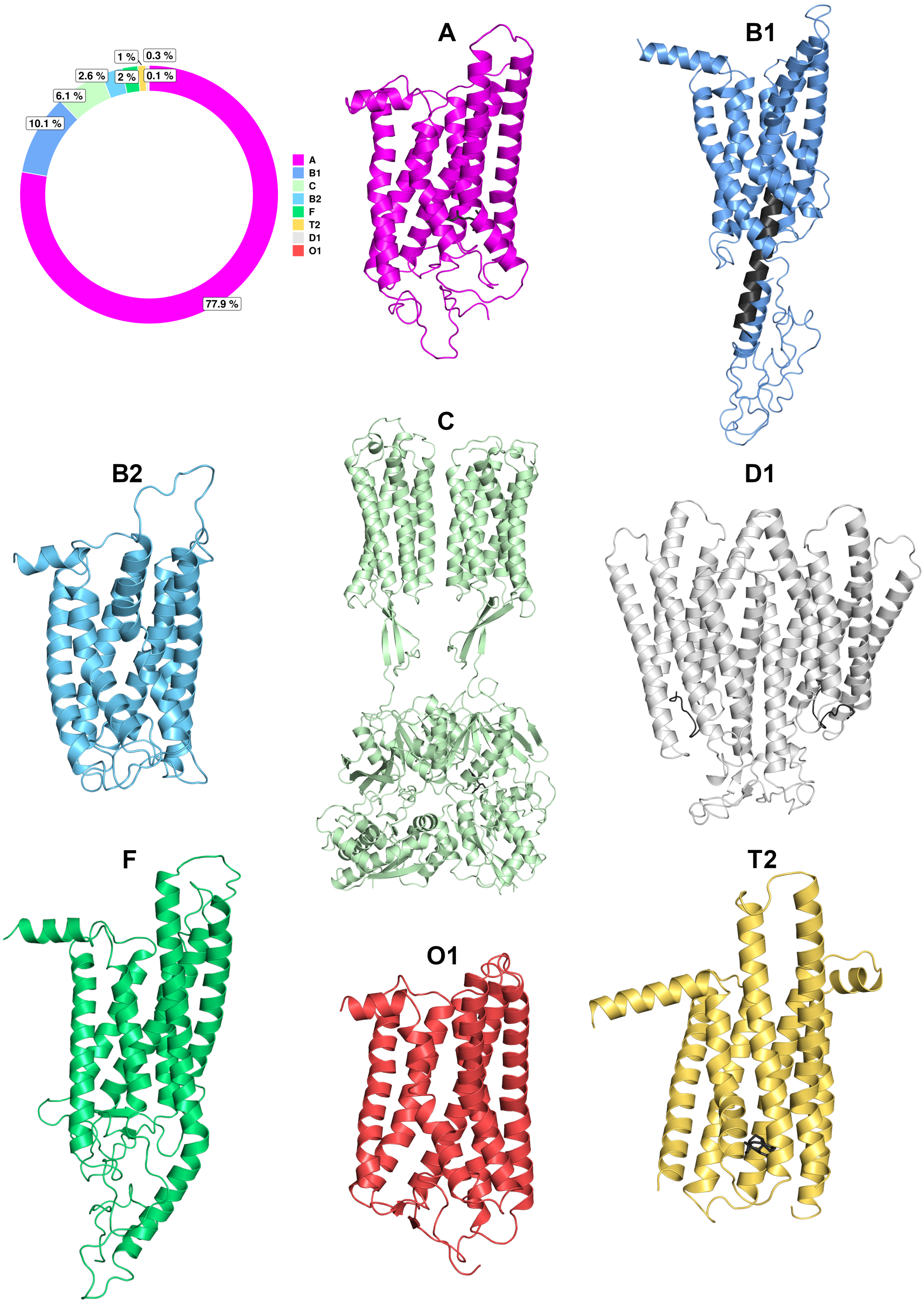

Figure S2. Representative structures of the different GPCR classes. The donut chart shows the distribution in the structural dataset of GPCRs from the different classes. Representative active-state structures from each class are shown as well in cartoons. The PDB codes are: class A, 6OYA; B1, 6WZG; B2, 7WU5; C, 7EB2; D1, 7AD3; F, 6OT0; O1, 8F76; T2, 7XP6.

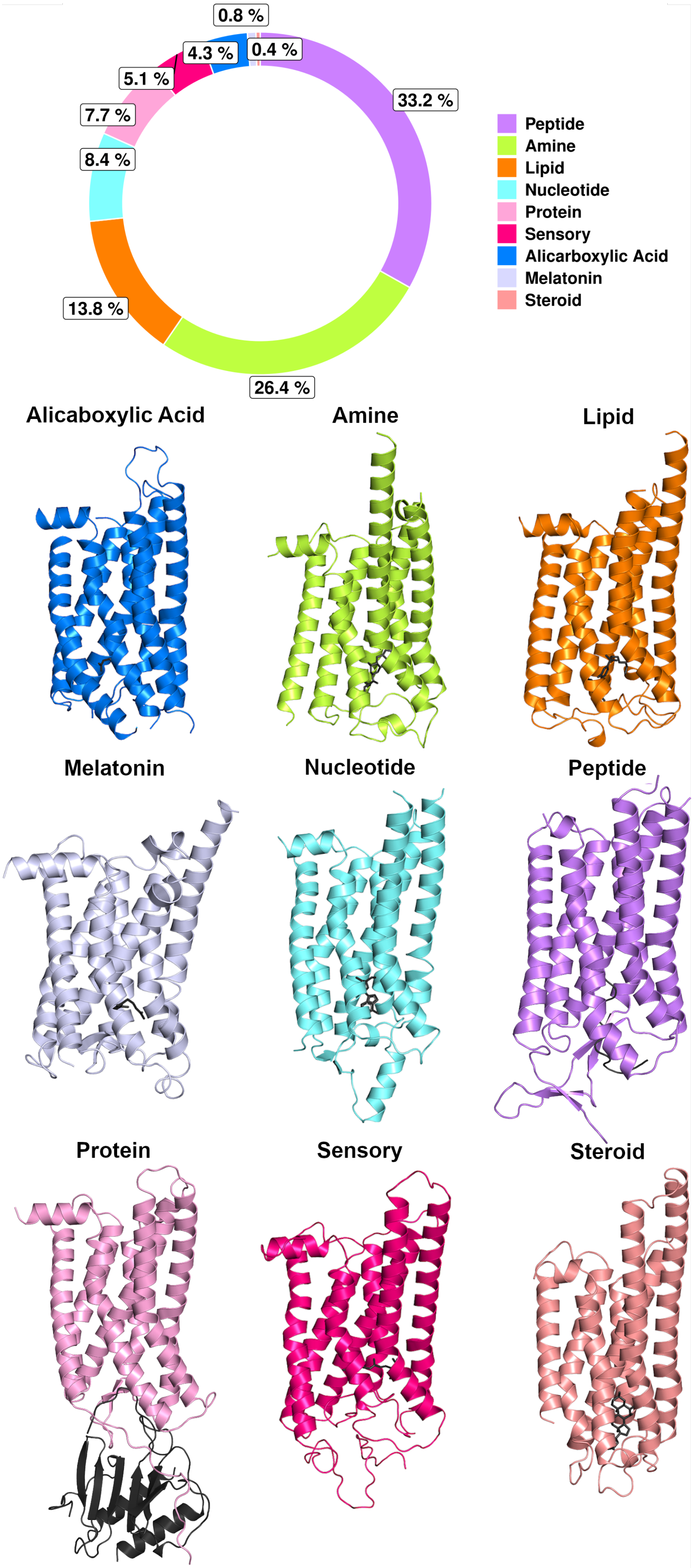

Figure S3. Representative structures of the different sub-families of class-A GPCRs. The donut chart shows the distribution in the structural dataset of class-A GPCRs from different sub-families. Representative structures from each sub-family are shown as well in cartoons. The relative PDB codes are: alicarboxylic acid, 8YKW; amine, 8FYT; lipid, 8GUR; melatonin, 7VGY; nucleotide, 7LD4; peptide, 9JFY; protein, 8XX6; sensory, 6OYA; and steroid, 7CFN.

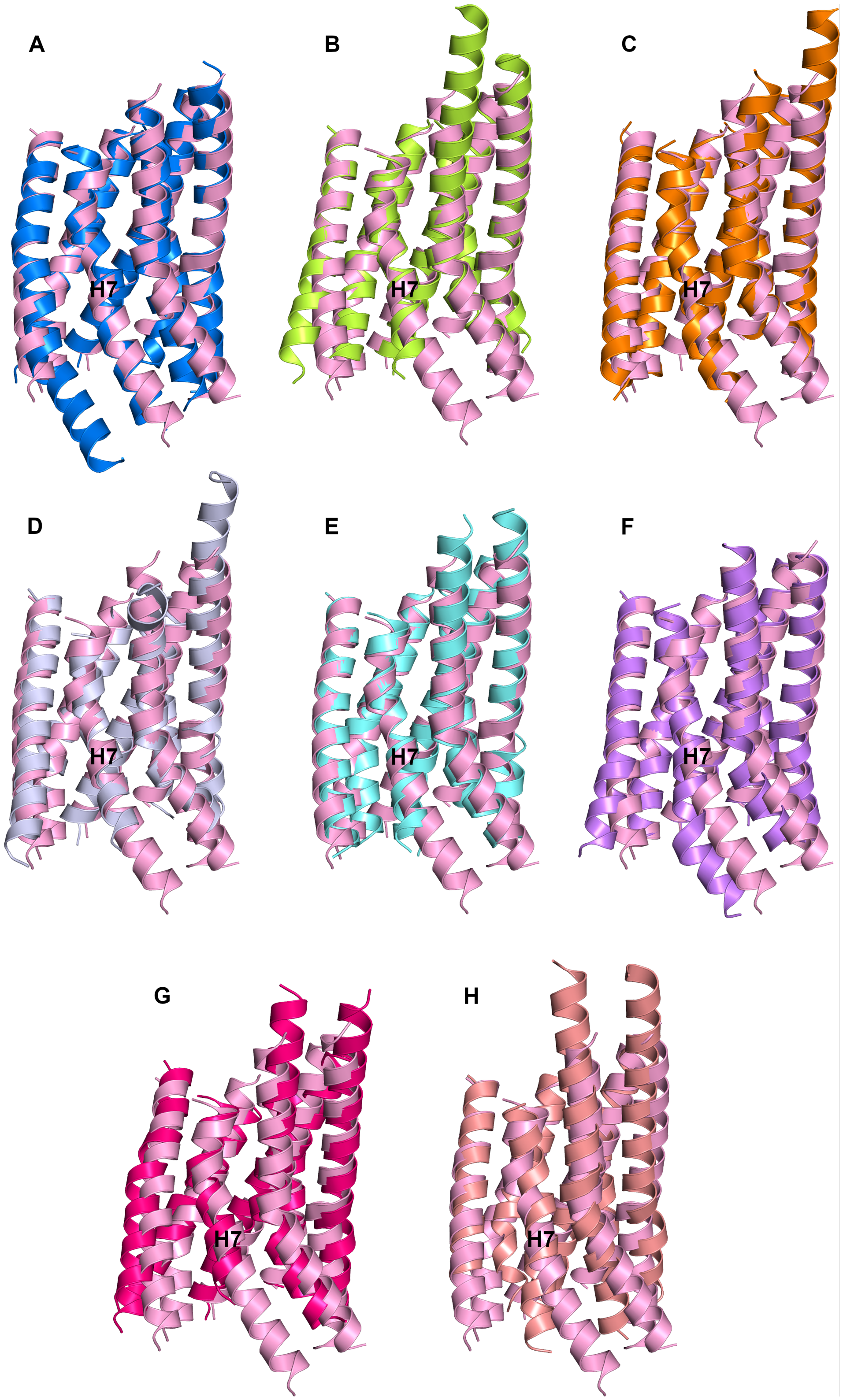

Figure S4. Comparisons of the helix bundles. The helix bundle of the representative structure of protein GPCRs (8XX6) is superimposed to those of **A.** Alicarboxylic acid (8YKW), **B.** amine (8FYT). **C.** lipid, (8GUR), **D.** melatonin (7VGY), **E.** nucleotide (7LD4), **F.** peptide (9JFY), **G.** sensory (6OYA), and **H.** steroid (7CFN) GPCRs. Receptors are seen in a direction perpendicular to the bundle main axis.

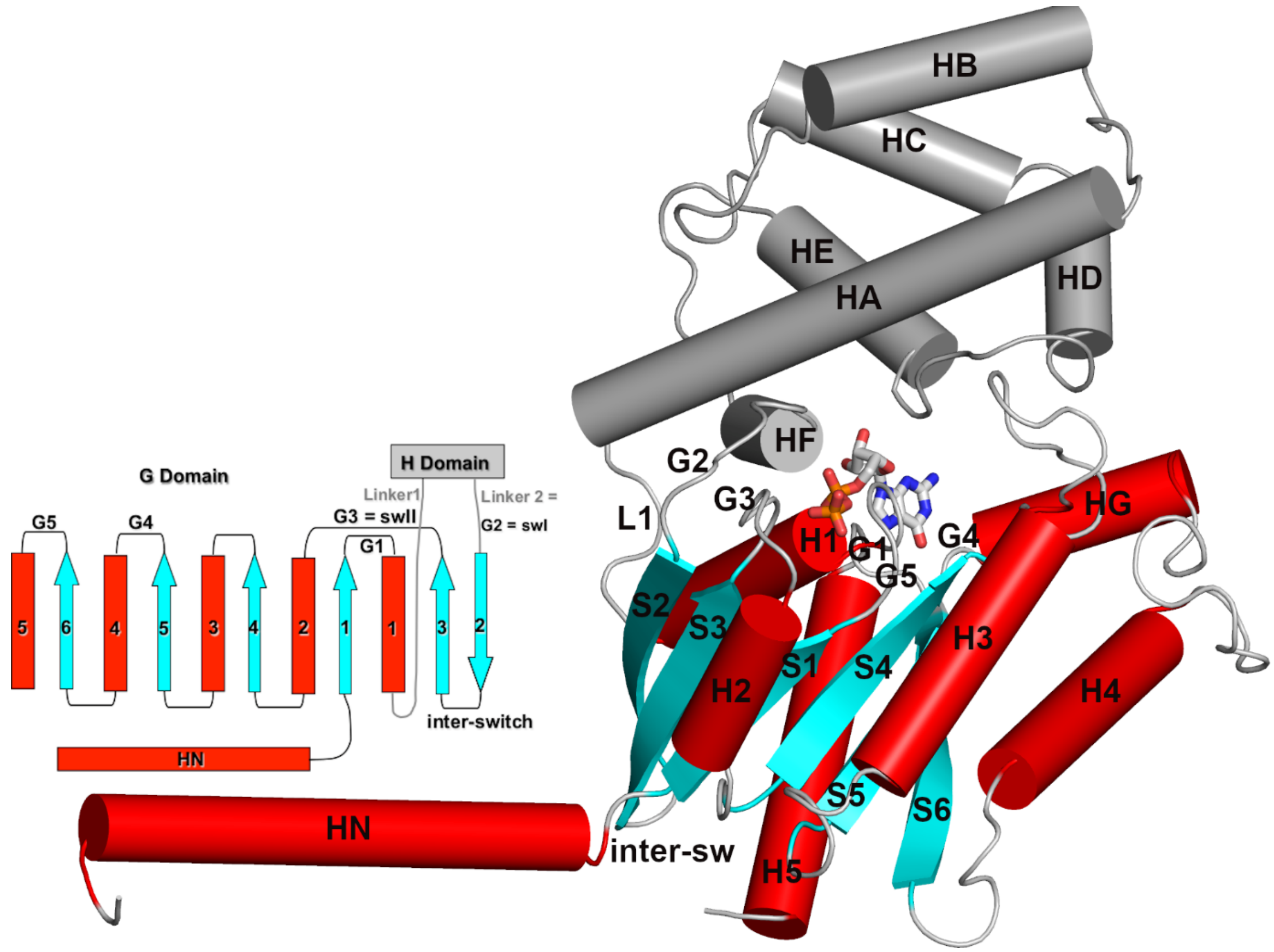

Figure S5. Structural features of the heterotrimeric G protein. The topology diagram and the 3D-structure of GDP-bound heterotrimeric G protein are shown. Helices and strands are, respectively, red and cyan. The different secondary elements are labeled according to the scheme adopted by the GPCRdb (https://gpcrdb.org/). The G domain holds a Rossmann fold, characterized by a 3-layer(αβα) sandwich architecture. The 3-layer(αβα) sandwich is made of five parallel strands forming a sheet packed against five helices: H1 and H5 on one side and H2-H4 on the other side. Two linkers connect the G domain to the helical (H) domain, which is an orthogonal bundle of six helices (HA-HF). The nucleotide docks into a binding site contributed by the G.s1h1, G.h1s2 (G.hfs2 in the Gα proteins), G.s3h2, G.s5h4, and G.s6h5 loops (i.e., G boxes 1-5 (G1-G5). Sequence conservation over the Ras superfamily resides just in the G boxes, also defined as ultraconserved regions. G1, originally termed as the Walker A motif (motif GxxxxGKS/T), is also called P-loop (phosphate-binding loop). The latter, in fact, connects the phosphates through main-chain NH groups and lysine side chain. G2 is also called switch I (swI or G.h1s2 loop (G.hfs2 loop in the Gα proteins)) and G3 is part of the switch II (swII made by the G.s3h2 loop plus the G.H2 helix). The β-hairpin between the two switches is called inter-switch. The polypeptide chain leaves the G domain and enters the H domain through the h1ha loop or linker 1 (L1) and successively leaves the H domain and goes back into the G domain through the hfs2 loop (swI or L2). The interface between G and H domains constitutes the nucleotide-binding cleft.

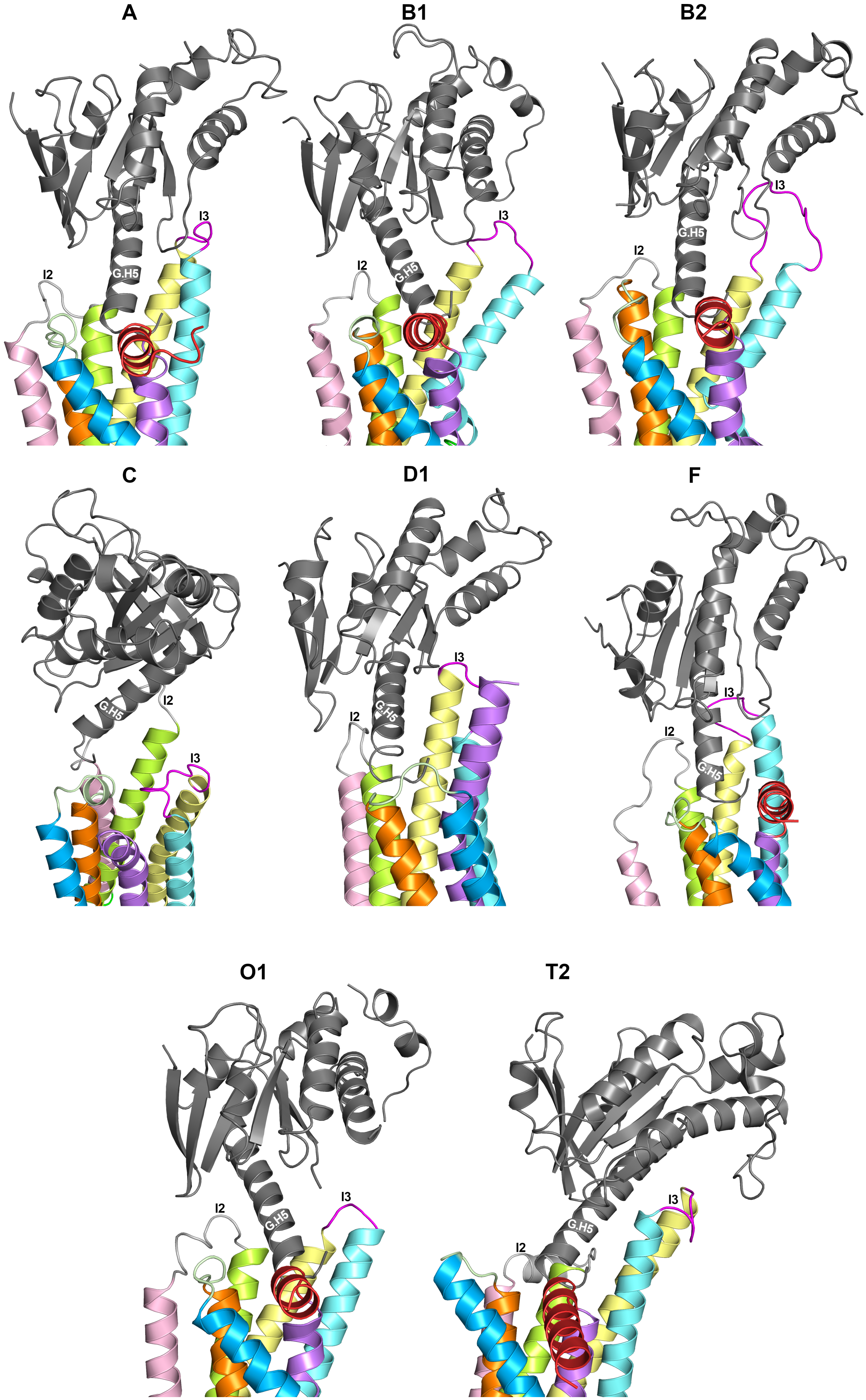

Figure S6. Receptor-G protein interfaces The interfaces between the G domain of the G protein and the cytosolic regions of representative GPCRs from different classes is shown (class A, 6OYA; B1, 6WZG; B2, 7WU5; C, 7EB2; D1, 7AD3; F, 6OT0; O1, 8F76; T2, 7XP6). The G.HN helix is not shown.

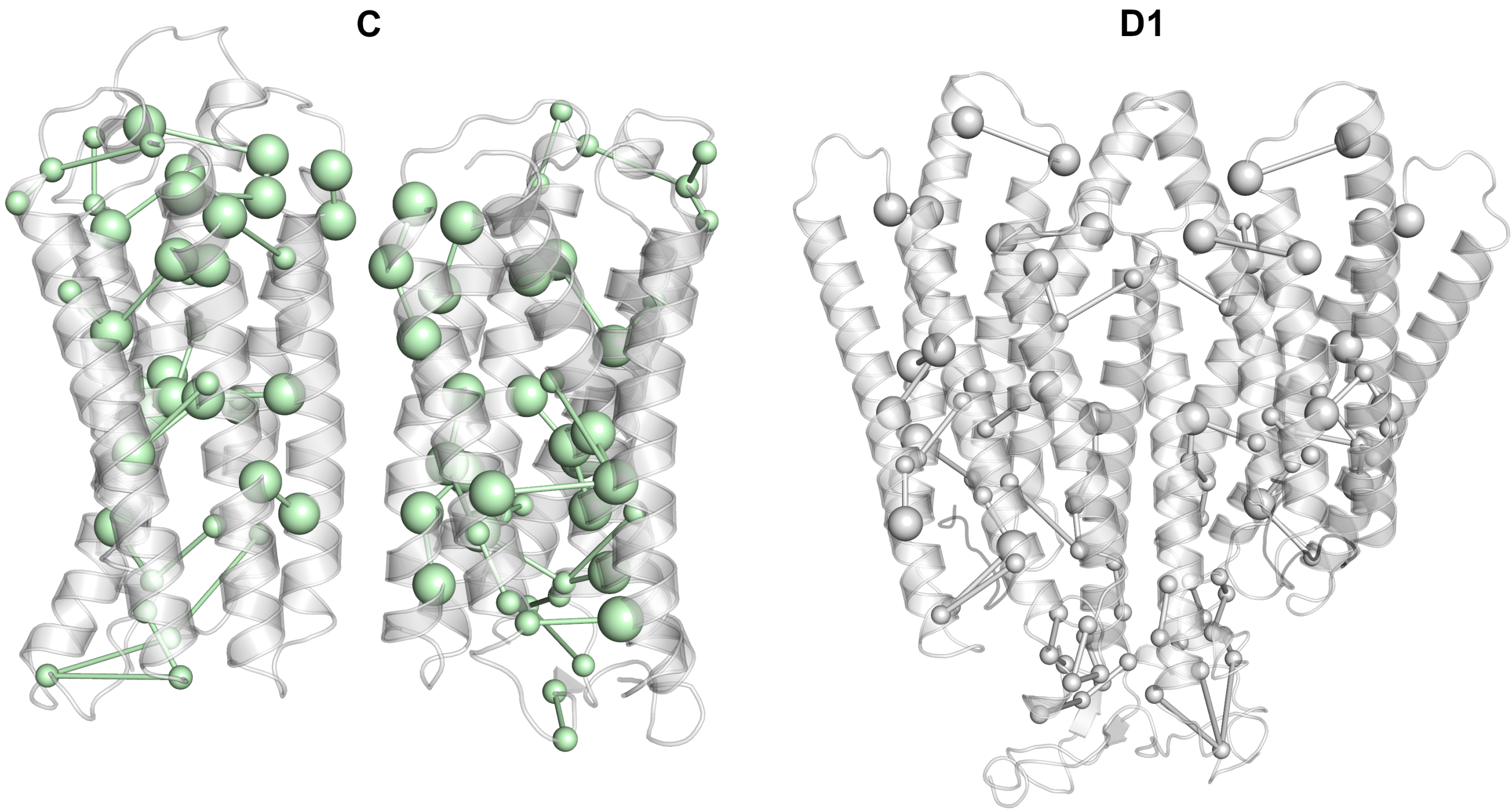

Figure S7. Specific links in dimeric classes. The specific links of dimeric classes C and D1 are shown. For class C, only the protomer on the left site is bound to the G domain of the G protein, whereas for class D1, the protomer on the right site is bound to the G domain and the protomer on the left side is bound to the H5 helix of the G protein. The biggest spheres concern the highly conserved nodes (characterized by ConSurf conservation grades ≥8). The N-terminus and E2 of class C is not shown.

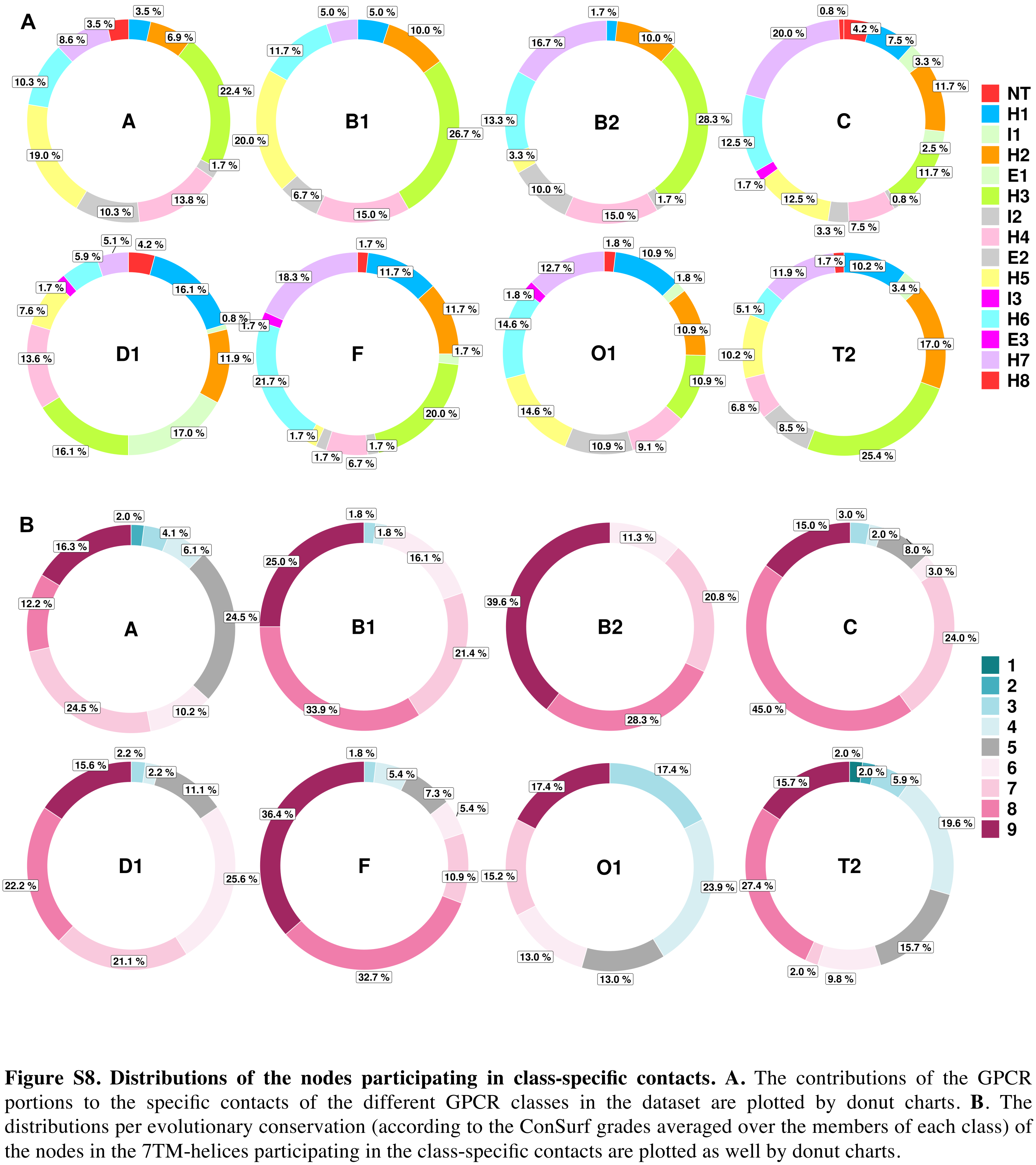

Figure S8. Distributions of the nodes participating in class-specific contacts. A. The contributions of the GPCR portions to the specific contacts of the different GPCR classes in the dataset are plotted by donut charts. **B**. The distributions per evolutionary conservation (according to the ConSurf grades averaged over the members of each class) of the nodes in the 7TM-helices participating in the class-specific contacts are plotted as well by donut charts.

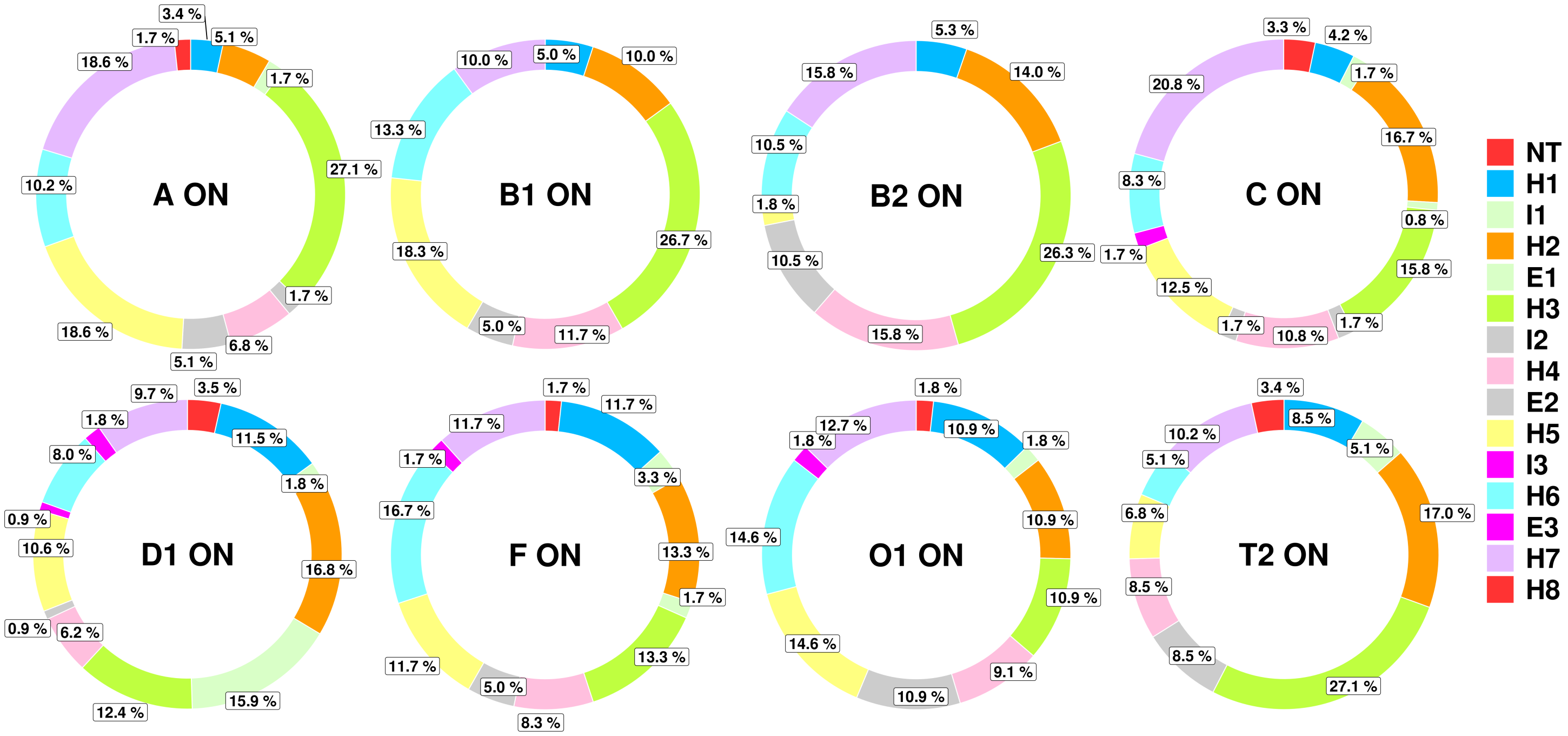

Figure S9. Distributions of the nodes participating in class-specific contacts in the active states. A. The contributions of the GPCR portions to the specific contacts of the different GPCR classes in the ON state sub-sets are plotted by donut charts.

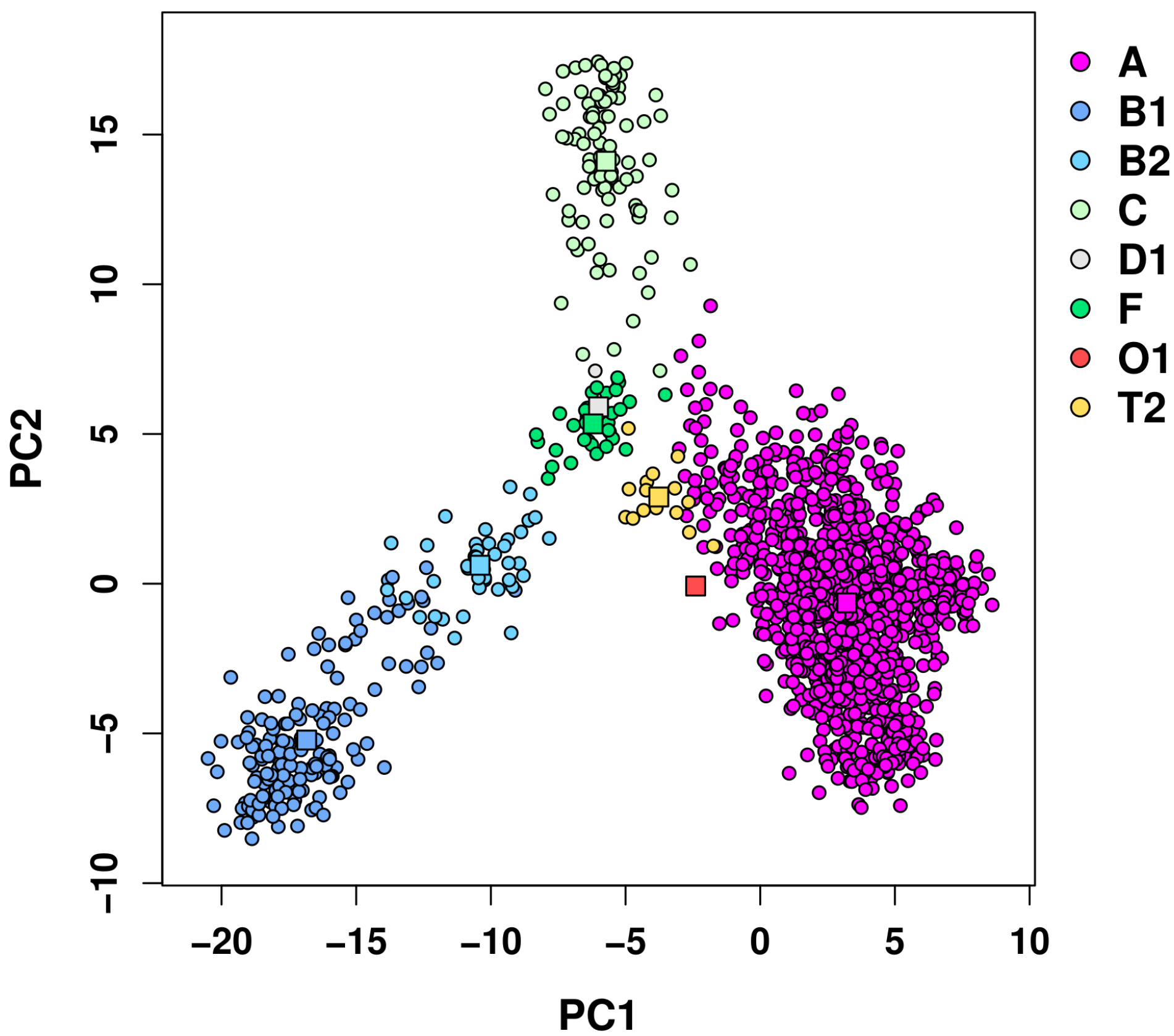

Figure S10. Specific contact-based separation of GPCR classes. The plot of the first two principal components employing the pairwise differences of class-specific contact average strengths as variables are shown.

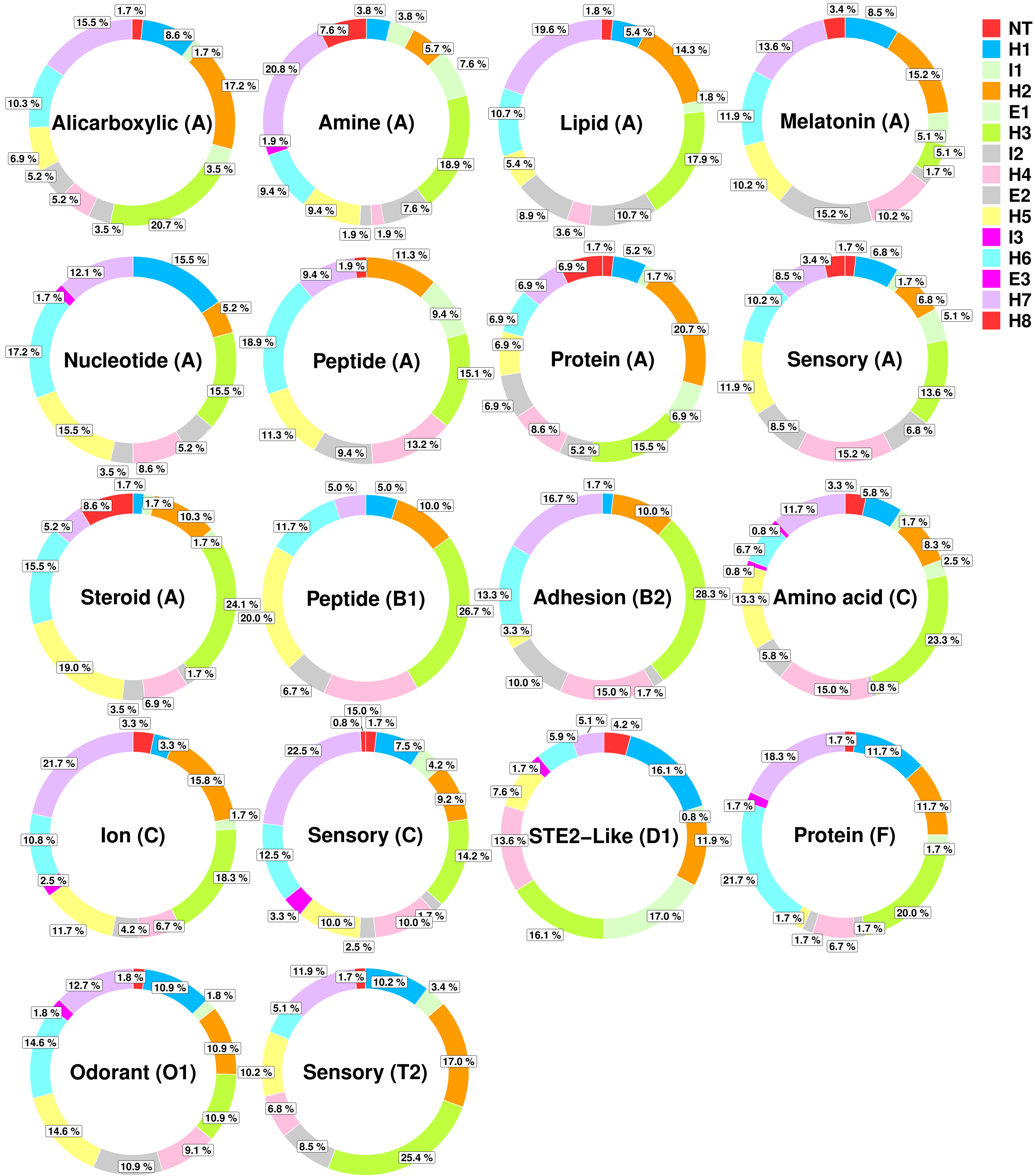

Figure S11. Contribution of the different receptor portions to sub-family-specific contacts. The contributions of the GPCR portions to the specific contacts of the different GPCR sub-families in the dataset are plotted by donut charts.

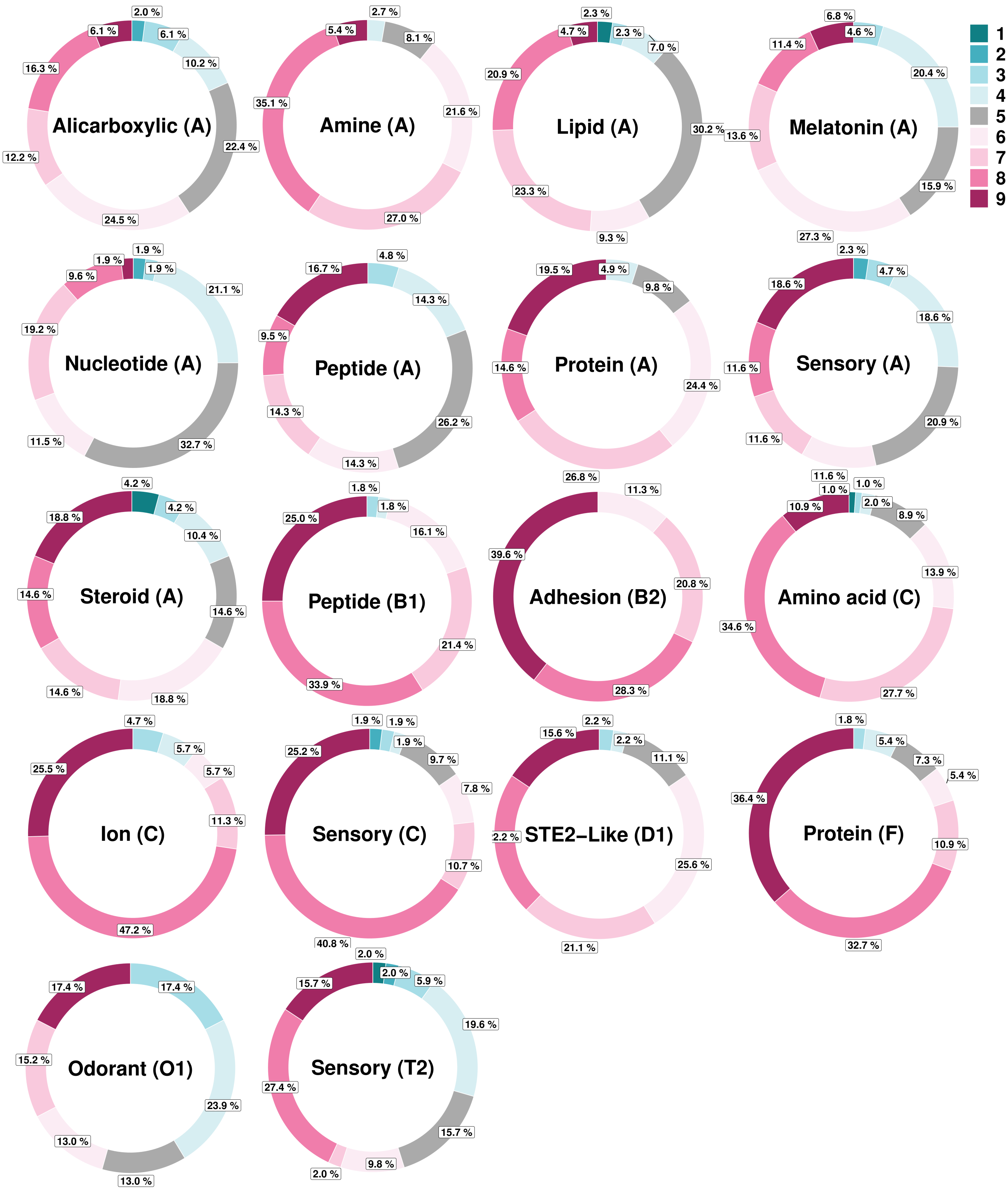

Figure S12. Distributions of the nodes participating in sub-family-specific contacts. A. The distributions per evolutionary conservation (according to the ConSurf grades averaged over the members of each considered sub-family) of the nodes in the 7TM-helices participating in the sub-family-specific contacts are plotted by donut charts.

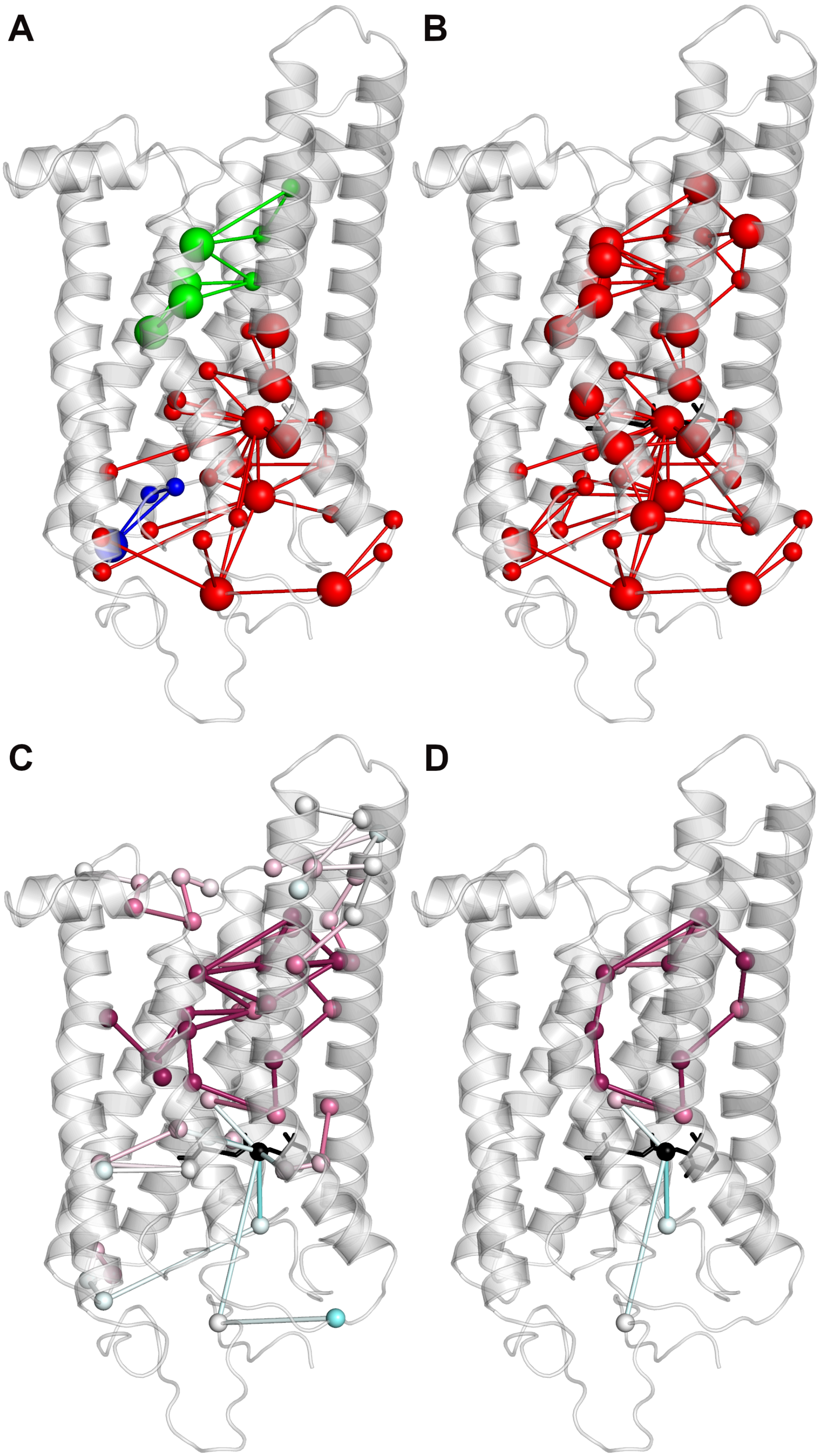

Figure S13. Communities and global communication pathways of class-A ON state. Node communities (**A** and **B**) and global communication pathways (**C** and **D**) have been computed on the receptor-G protein complexes of the active-state set by excluding (**A** and **C**) and including (**B** and **D**) the G protein from calculation. In the latter case, only the receptor is shown. As for the communities, the biggest spheres are nodes with hub behavior. In the absence of the G protein, three communities form (red: 35 nodes and 44 links; green: 7 nodes and 9 links; and blue: 3 nodes and 3 links). In the presence of the G protein a single community forms on the receptor, made of 54 nodes and 77 links. In the global metapaths (C and D), nodes are colored according to the ConSurf conservation grades, whereas links are colored according to the average ConSurf grades of the connected nodes.

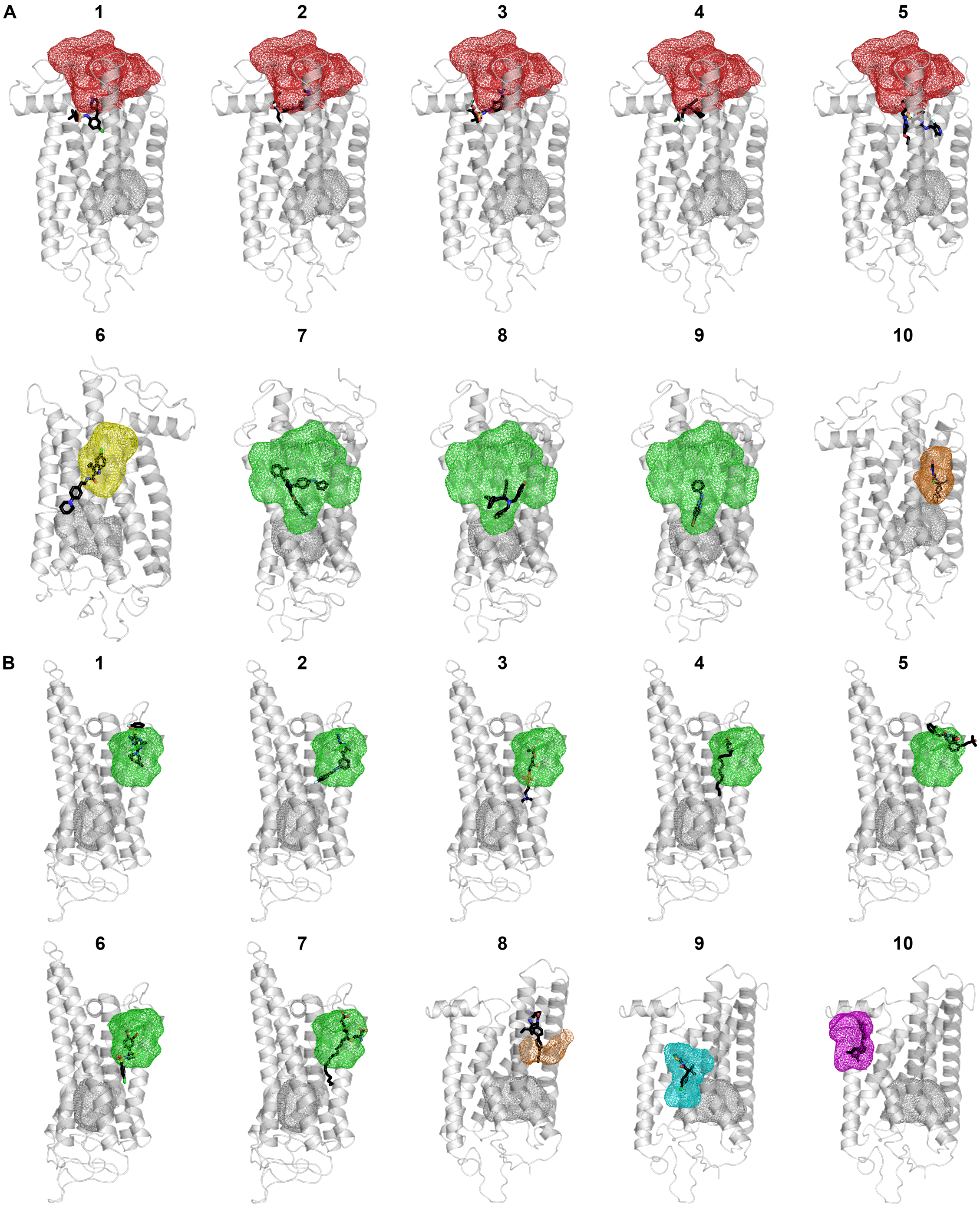

Figure S14. Match between predicted cavities and allosteric modulator sites. The predicted allosteric cavities are mapped into the structures of OFF (1U19) and ON (3OYA) rhodopsin. The allosteric cavities are colored according to their location: G protein binding site (G protein, red), outer faces of H1, H2, and H4 (H1,2,4, yellow), H1, H7, and H8 (H1,7,8, magenta), H3, H4, and H5 and interconnecting loop (H3-5, green), H5 and H6 (H5,6, orange), and H6 and H7 (H6,7, cyan). The orthosteric cavity is grey. The cryoEM structures of a number of GPCRs bound to small negative or positive allosteric modulators have been, respectively, superimposed to those of OFF or ON rhodopsin structures, displaying only the allosteric ligands in sticks colored by atom-type (carbon atoms are green or magenta depending on the color of the cavity). Those compounds fit the predicted allosteric cavities. In detail, in the OFF states (**A**), **1**) vernicion bound to CCR9 (5LEW), **2**) PubChem153466996 bound to CCR2 (6LFL), **3**) Cmp2105 bound to CCR7 (6QZH), **4**) CCR2-RA-[R] bound to CCR2 (5T1A), and **5**) PubChem168510218 bound to GPR61 (8TB7) recognize the allosteric cavity overlapping with the G protein binding site; **6**) ORG27569 bound to the CB1R (6KQI) recognizes the H1,2,4 cavity; **7** and **8)** avacopan or NDT9513727 bound to the C5aR (6C1R and 6C1Q, respectively), and **9**) AS408 bound to the β2AR (6OBA) recognize the cavity H3-5; and **10**) SCH546738 bound to CXCR3 (8HNN) recognizes the H5,H6 cavity. As for the ON states, **1** and **2**) compound-187 or AZ1729 bound to FFA2R (9NS9 and 9CM7, respectively), **3**) lysophosphatidylcholine (LPC) bound to GPR4 (9JCO), **4**) docosahexanoic acid (DHA) bound to FFA1R (9K1C), **5**) LY3154207 bound to D1R (7LJD), **6**) BMS986122 bound to MOP (8Y72), and **7**) cysteinyl leukotriene E4 (LTE4) bound to GPR99/OXGR1 (8YYX) recognize the cavity H3-5; **8**) (1R, 2R)-2-PCCA bound to GPR88 (7EJX) recognizes the cavity H5-6; **9**) 4-CMTB bound to FFA2R (8Y6W or 9CLW) recognizes the cavity H6-7; and **10**) PSB-16671 bound to GPR84 (10JB) recognizes the cavity H1,7,8.

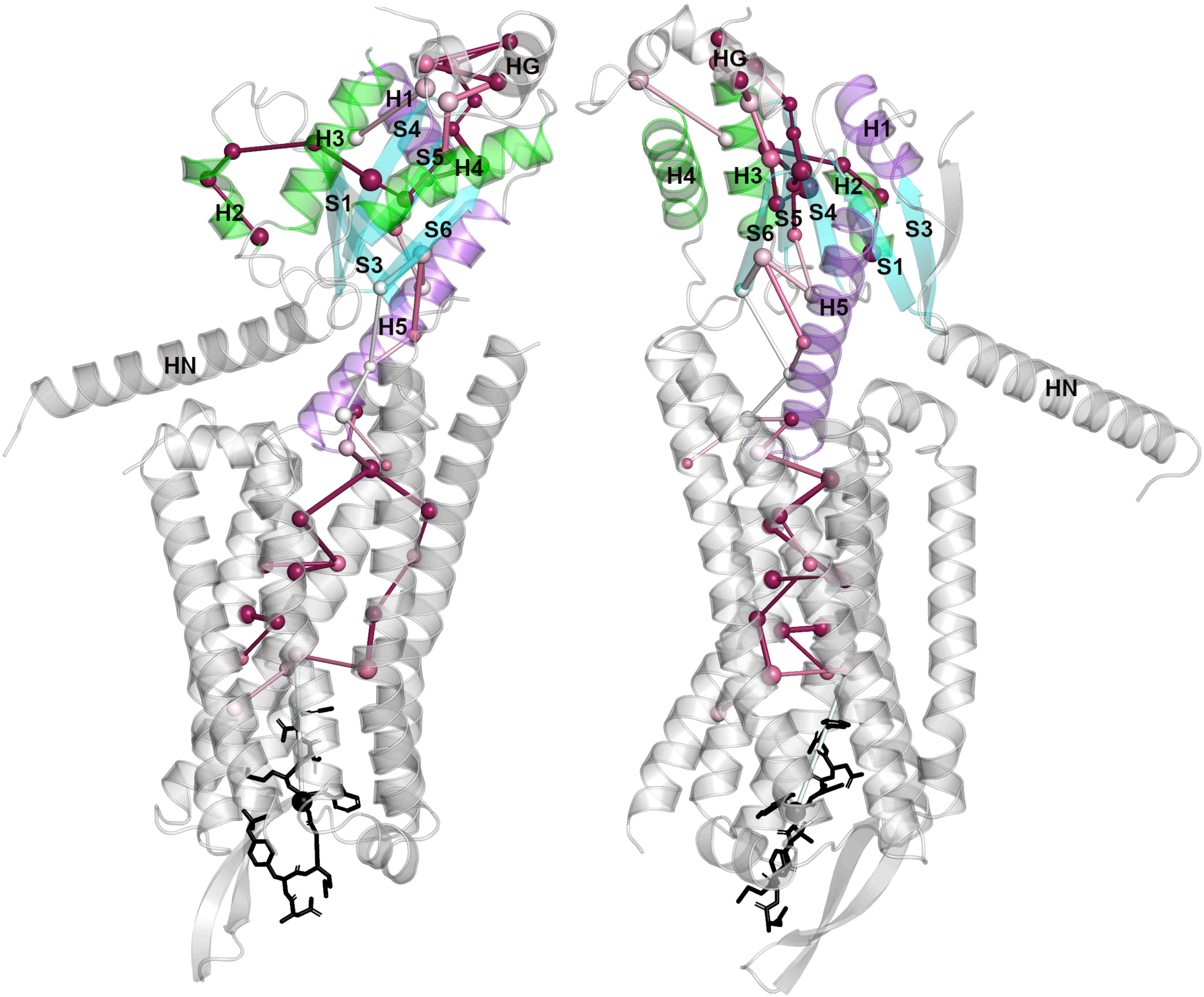

Figure S15. Consensus metapath of class-A Gi. The metapath, mapped on the structural complex between the CCKA receptor and Gi (PDB: 7EZH), is colored by ConSurf grades. The parallel sheet in the G domain are cyan, the two helices (G.H1 and G.H5) forming the interface-1 with the sheet are violet, whereas the three helices (G.H2, G.H3, and G.H4) forming the interface-2 with the sheet are green.

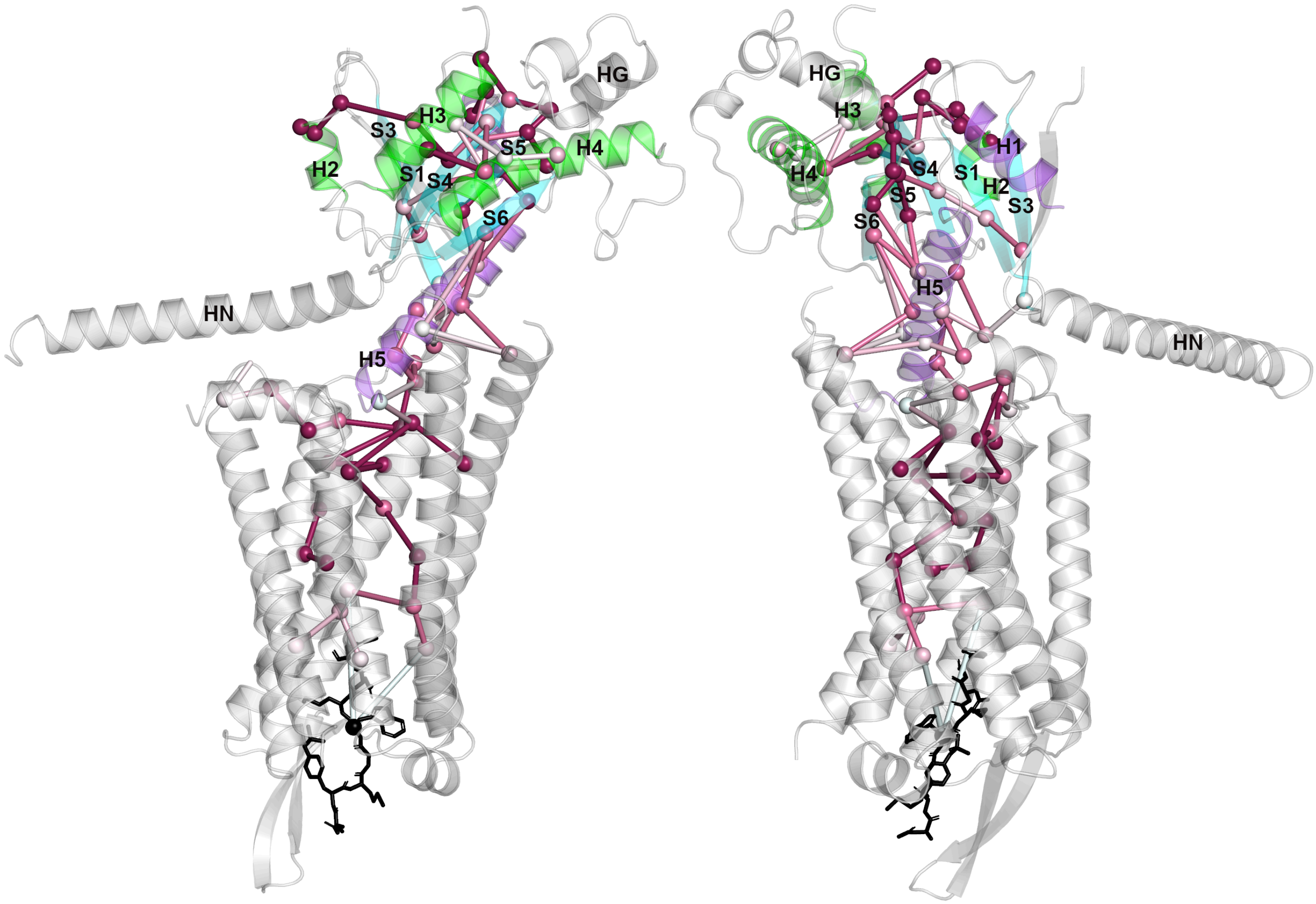

Figure S16. Consensus metapath of class-A Gs. The metapath, mapped on the structural complex between the CCKA receptor and Gs (PDB: 7MBX), is colored by ConSurf grades. The parallel sheet in the G domain are cyan, the two helices (G.H1 and G.H5) forming the interface-1 with the sheet are violet, whereas the three helices (G.H2, G.H3, and G.H4) forming the interface-2 with the sheet are green.
